# Marriage, parenthood and social network: Subjective well-being and mental health in old age

**DOI:** 10.1371/journal.pone.0218704

**Published:** 2019-07-24

**Authors:** Christoph Becker, Isadora Kirchmaier, Stefan T. Trautmann

**Affiliations:** 1 Alfred-Weber-Institute for Economics, University of Heidelberg, Heidelberg, Germany; 2 Department of Economics, Tilburg University, Tilburg, The Netherlands; University of Zurich, SWITZERLAND

## Abstract

Parenthood, marital status and social networks have been shown to relate to the well-being and mental health of older people. Using a large sample of respondents aged 50 and older from 16 European countries, we identify the associations of well-being and mental health with family status. Making use of detailed social network data of the respondents, we also identify how different social support networks correlate with the well-being and health indicators. We observe positive associations for all network types, over and beyond any direct associations of family status with well-being. Results suggest that non-residential children are important providers of social support for their parents at older age.

## Introduction

The link between family status (marital status and parenthood), well-being, and mental health is widely discussed in academic and popular discourses. Evidence suggests that being married or living with a partner can have a positive effect on life satisfaction [1] and is associated with higher well-being, better mental health and fewer depressive symptoms in old age [2–4].

Parenthood, on the other hand, does not appear to be associated with enhanced mental health [5–7]. The risk of depression is especially pronounced for women with parenting stress and poor physical health, but less pronounced for those being supported by the partner [8]. Repeated cross-sectional data on US parents and non-parents shows a gap in subjective well-being between these two groups, which, however, becomes smaller over the period 1973 through 2008 due to decreased happiness of non-parents [9]. A cross-country comparison finds only weak associations between life satisfaction and having children, with unclear direction [1]. However, there is also evidence that the relationship between children and well-being becomes more positive for older respondents [1,10]. Depending on the life-cycle stage, the aspects of parenthood may thus differ, suggesting that the positive aspects of parenthood dominate when getting older. Amongst others, the role of children as a form of social support may become important in the later stages of a person’s life [10].

But what constitutes social support? One of the most cited definitions stems from Cobb [11], describing social support as “information leading the subject to believe that he is cared for and loved, esteemed and a member of mutual obligations”. The U.S. National Cancer Institute [12], defines social support as “a network of family, friends, neighbors, and community members that is available in times of need to give psychological, physical, and financial help”. In general, a social network consists of a “set of actors and the ties amongst them” [13], while the term social support further describes the quantity and quality of these ties from an individual perspective. While there exist multiple definitions of social support, most of them encompass factors for the size and structure of the network, as well as including measures for physical distance to other network members, length of the relationships, frequency of contact or function of each relationship [14]. Evidence suggests that such social support networks are related to less loneliness and more happiness [15,16] and act as important buffer against stressful events [11,14].

While results on parenthood might be controversial and depend on the age of the studied population, there is widespread agreement that social support is associated with higher life satisfaction, and that social networks are an important factor for well-being [17]. Bringing these two branches of the literature together, we aim to shed light on the link between a person’s family status, the resulting characteristics of their social networks, and their well-being and mental health, using a large sample of 55.000 middle-aged and older adults from 16 European countries. This sample was taken from the Survey of Health, Ageing and Retirement in Europe (SHARE). For people aged 60 and older in Mediterranean and non-Mediterranean countries in the first wave of the data set, there is some evidence that the number of residential children is associated with more depressive symptoms [18]. We aim to expand and generalize these findings using recently collected, detailed network data, across European countries. Parenthood, marital status and different types of social networks might help to sustain well-being and mental health in old age. Our objective in the current study is to analyze the role of marital status, parenthood and social networks deriving from these family backgrounds as potential sources of social support, for well-being and mental health in old age. We consider four distinct measures, used in different fields such as economics or psychology, to obtain a comprehensive picture of well-being and mental health. These are the CASP-12 scale for quality of life, the EURO-D scale for depressive symptoms, and one question each for life and social support network satisfaction.

We use the full range of the SHARE data set, which includes people aged 50 and older. At this point in the life cycle, parents may have resident children, children living away from home, and grandchildren, allowing us to separate their associations with well-being. We use network composition measures in order to determine network types, and control for network size and relational dynamics separately. Additionally, we calculate the network types for each country separately, taking cultural differences in network compositions into account.

Based on the current literature we test the following three hypotheses for the well-being and mental health of people aged 50 and older: i) A positive association with being married, ii) a positive association with the number of children and grandchildren not living at home, and iii) a positive association with having a strong social network implied by family background. We proceed as follows: Section 2 describes the data used and our methods to measure well-being, mental health and the characteristics of social support networks in detail. In Section 3 we present the results of our analysis. We first analyze the association of family status with well-being and mental health measures without taking the social network into account. We then take the network composition as criterion variables and use hierarchical clustering to determine social network types which differ mainly in their main source of social support. We then assess the relationship between the resulting social support network types and outcome measures, controlling for family status, network size, and relational dynamics. Section 4 discusses our findings, and Section 5 provides concluding remarks.

## Data and methods

### Respondents

We use data from the cross-national panel database Survey of Health, Ageing and Retirement in Europe (SHARE), release 6.0.0., managed by the Munich Center for the Economics of Aging, Max Planck Institute for Social Law and Social Policy [19–21]. The cross-national panel database provides extensive data on health and socio-economic status. The target population is people of age 50 or older having their regular domicile in the respective country. Current partners are interviewed regardless of their age. We make use of SHARE wave 4 [22] that was administered between 2010 and 2012 in 16 European countries, and includes a module on social network. We update missing constants with data from waves 1 and 2 [23,24]. We include respondents age 50 and older not living in a nursing home. The number of respondents differs by country. Over all countries, there are about 55.000 observations available. For an overview of the total number of observations of each country, see the supporting S1 Table.

### Demographic factors

The SHARE data set contains detailed data on demographics. Summary statistics of all demographic variables used in the analyses can be found in S2 Table. The demographic factor of interest is the family status, which we measure by the marital status, total number of children, children living at home, and grandchildren. Over all countries 70% of the respondents are married and 91% have children.

The marital status of each respondent is classified into the categories (1) married and living together with spouse, (2) registered partnership, (3) married and living separated from spouse, (4) never married, (5) divorced, (6) widowed. For the regression analysis we construct the dummy variables *married* which takes the value of one if the respondent is married or in a registered partnership, the dummy variable *divorced*, which takes the value of one if the respondent is divorced or living separated from spouse, and a dummy variable *widowed*. We include respondents living separately from their spouse in the dummy *divorced*, as living separately is often a preceding step to a divorce.

Parenthood is measured by the number of children alive and the number of resident children, including fostered, adopted and stepchildren. We define the four-category measure *children* with categories no children, one child, two children, and three or more children, and create the respective dummy variables for each category. We further construct the variables *resident children* and *grandchildren* which report for each respondent the number of children living with the family and the number of grandchildren.

Further demographics are used as controls. The set *Controls A* consist of gender, age (of the respondent at the time of the interview), age squared, and a dummy variable indicating the country of residence of the respondent to control for cultural differences. The set *Controls B* additionally includes dummies for urban character of residence, being employed, self-employment, level of education according to the international classification of education ISCED-97 [25],an indicator for the average monthly household income, and the aforementioned dummies for divorced and widowed. In SHARE wave 4, each household respondent is asked to state the overall after-tax income of the entire household in an average month of last year. If a respondent refuses to answer, the interviewer asks whether the respondent earns more, less or approximately the amount in certain bracketed values, which represent country-specific 25^th^, 50^th^, and 75^th^ percentiles of the reported household incomes from SHARE wave 2. We use the information from the stated household income and the unfolding brackets and define four categories for the average monthly household income: (1) Low income [0 to 25^th^ percentile], (2) Middle income [25^th^ to 50^th^ percentile], (3) Upper middle income [50^th^ percentile to 75^th^ percentile], and (4) High income [75^th^ percentile and higher]. The boundaries of the intervals are the country-specific bracket values of SHARE wave 4 (details and summary statistics in S2 Table).

In order to control for health, we include a measure of self-assessed physical health (Would you say your health is: (1) poor, (2) fair, (3) good, (4) very good, and (5) excellent), and whether drugs for sleeping problems, anxiety or depression are taken.

### Well-being and mental health indicators

Well-being can be defined as the psychological balance point between individually available resources and challenges [26] and may be linked to many different aspects of life. In order to develop national well-being measures, the Office for National Statistics in the UK ran a public debate on the question through various platforms [27]. The three most frequent answers to the question “What things matter most in your life? What is Well-being?” were “Health”, “Having good connections with friends and relatives”, and “Job satisfaction (and economic security)” [28]. Many empirical studies report a link between socioeconomic status, quality and quantity of social contacts, and well-being [17]. In our study, we use a broad set of measures to map respondents’ well-being: a simple single-item question regarding life satisfaction; the CASP-12 multi-item quality of life scale; a single-item question on social support network satisfaction; and the EURO-D depressive symptoms scale. In the following, we will discuss the three measures in more detail. We also use measures of health, education, and financial status as controls in our analyses [29,30].

The first measure concerns a general feeling about the quality of life, the stated *Life satisfaction*. It is extracted by a single-item question in which respondents indicate on a scale from 0 (low satisfaction) to 10 (high satisfaction) how satisfied they are with their life. This scale has acceptable reliability and validity [31,32].

The second measure is the CASP-12, *quality of life* scale, which is designed to capture quality of life in old age [33]. Participants indicate for twelve statements whether they apply on a scale from 1 (often) to 4 (never). The twelve questions concern four dimensions of quality of life, control, autonomy, pleasure and self-realization, resulting in an aggregate index ranging from 12 (low quality of life) to 48 (high quality of life). We normalize it such that it ranges from 0 (low quality of life) to 10 (high quality of life).

The third measure concerns the stated *Network satisfaction*. Respondents indicate on a scale from 0 (low satisfaction) to 10 (high satisfaction) how satisfied they are with their social network. If respondents indicated that there is no person with whom they discuss matters or there is no one who is important to them, they were asked how satisfied they were with this fact.

The fourth measure is the EURO-D depression score [34]. It is an indicator for depressive symptoms and captures aspects of mental health in late life. It has been demonstrated to provide a valid comparison of depressive symptoms across European countries [35,36]. The EURO-D depression score is generated from questions on 12 dimensions: Depression, pessimism, suicidality, guilt, sleep, interest, irritability, appetite, fatigue, concentration, enjoyment, and tearfulness. Respondents are asked whether there is an indication for each of these dimensions. It results in an aggregate index ranging from 0 (not depressed) to 12 (very depressed). We normalize it such that it ranges from 0 (very depressed) to 10 (not depressed) and call it *Lack of depressive symptoms*.

**Fig 1:**
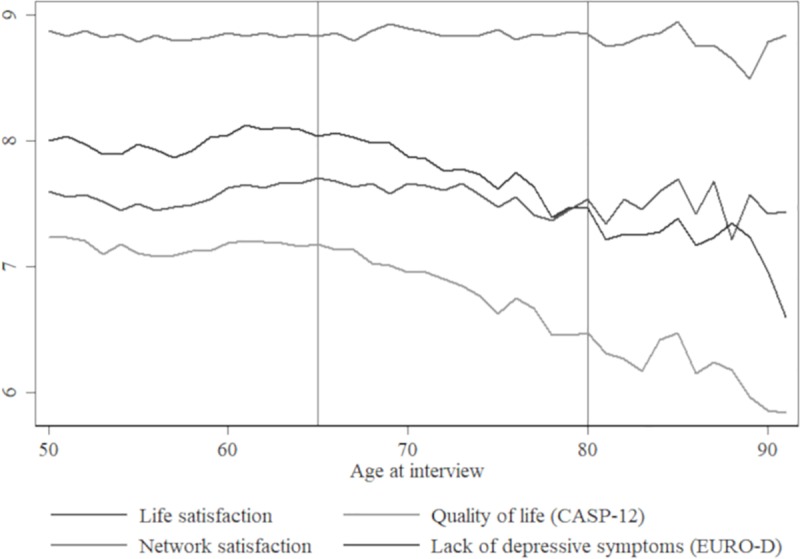
Average well-being and mental health measure. Average well-being and mental health measure for all ages from 50 to 90 years. After age 91 the number of available observations drops to less than 50.

Fig 1 presents the average of the well-being measures at each age until 91 (see S1 Fig for the age distribution). While network satisfaction and life satisfaction remain relatively stable, the quality of life index and lack of depressive symptoms index decline beyond age 65. The graphs for male and female respondents are rather similar, except for the lack of depressive symptoms index; male respondents have on average a 0.73 points higher index (p<0.01, Mann-Whitney-U test; S2 Fig).

### Social support networks

A social support network can be characterized by its size and composition (percentage of partner, children, other relatives, and friends in the network) and relational dynamics. In Wave 4, the SHARE respondents are asked to answer questions about their social support network along the dimensions (1) size, (2) relationship, (3) contact frequency, (4) proximity, and (5) closeness.

In order to identify the members of their *social support network*, the respondents were asked to mention the name of persons with whom they discuss important matters. The total number of persons in the social support network is its *size*. It is possible to mention up to seven persons, however this boundary is only mentioned if it is reached. Only 3% of the respondents reach this boundary. Most respondents state one, two or three persons as members of their social support network (28%, 25%, and 20% of the respondents, respectively). Evidence suggests that the number of network members is positively linked with life satisfaction [37], but that in old age the network is reduced to members with close contact [38]

The *composition* of a network refers to the relationship type between each member. A person who has daily contact with two children and a person who has daily contact with two friends have a social support network of equal size and contact frequency, however, they have a different main source of social support. In a meta-analysis, Pinquart and Sörensen [17] provide evidence that the quantity of social contacts with friends is more strongly related with subjective well-being than the quantity of social contacts with family. They argue that friends are voluntary relationships, and they are typically members of the same age group or share similar preferences. Still, especially in older age, spouses and children are a crucial part of networks. Later in life, parents desire open communication, but low interference in each other’s lives thereby maintaining independence in old age and minimizing intergenerational conflicts [39]. Brandt et al. [40] analyzed the type of support between older parents, their children and professional providers. They found that children play a central role in providing help for their parents in the household and with paperwork. In Southern Europe, they are more likely to also take over regular medical care. There can also be differences within family structures. Shanas [41] provides evidence that the immediate family (partner and children) is the major social support during illness, and the extended family (children, siblings, and other relatives) is the tie to the community.

There are different ways to determine these different types of networks. One way is to construct network types, in which people are similar along family status (e.g. marital status, number of children and close relatives) and network measures (e.g. number of close friends, frequency of contact with family and friends, and frequency of attending social events). Commonly, there are four to five network types identified which differ in their relationship with well-being and mental health [15,42–44]. Another way to determine network types is to use only characteristics of the social network as criterion variables and control for family status separately. Litwin and Stoeckel [16] use size, composition and relational dynamic measures of the network and identify six networks (Spouse, Children, Spouse and Children, Other Family, Friend, and Other). They show that the network types are related differently to quality of life and that the frequency of the network types differs across European countries. We will follow a different approach, using only network composition to determine network types (i.e., the relative relevance of spouses, children, friends and others). We calculate network types for each country separately, taking cultural differences in network compositions into account. We chose this approach, because we want our network types to be directly linked to the family background variables whose associations with well-being we are interested in, and we control separately for contact frequency, emotional closeness and geographical proximity. In this way, we aim to identify how, for example, a Children network relates to well-being compared to a Friends network, conditional on controlling for frequency and closeness etc.

We classify the possible relationships into five categories: (1) Partner, (2) Children, (3) Other Relatives, (4) Friends, and (5) Others. Each of these categories comprises all types of relationships related to the category itself, i.e. the category Partner also includes the relationship “mother/father in law”. The *relationship share* of each category in the network of a respondent is measured by the sum of the occurrence of the category divided by the network size. For each respondent, the relationship shares of all relationship categories sum to one. We use the relationship share to determine country-specific support network types according to the main source of social support. The respondents who indicate that there is no person with whom they discuss important matters are excluded. For the remaining respondents, we use hierarchical clustering with the Ward [45] method to determine clusters which are similar with respect to the relationship shares. We choose to cut at six clusters and label them Partner, Children, Other Relatives, Family, Friends, and Diverse network. Using five clusters would not allow us to distinguish between the Friends and the Diverse network. Using more than six clusters does not provide an additional distinct network type for all countries for the five relationship categories used.

For each country, a cluster is labelled as Partner, Children, Other Relatives, Friends or Diverse network if the mean of the relationship share (averaged over all people in the cluster) of the category Partner, Children, Other Relatives, Friends and Other is higher in that cluster than in all other clusters, respectively. The labeling of the clusters would mostly be unaffected if it were instead determined by the highest mean relationship share (averaged over all people in the cluster) within a cluster, i.e., comparing across relationship type. Additionally, we include a cluster for Family networks. The Family network is the cluster with the highest sum of Partner share plus Children share plus Other Relatives share, excluding the clusters which are defined as Partner, Children, or Other Relatives network.

Apart from size and composition, a network is also characterized by relational dynamics such as geographical proximity, contact frequency and interpersonal closeness. Frequent contact with one’s children appears to be associated with less depressive symptoms, albeit irrelevant of geographical proximity [4]. Closeness with the support network member affects the quality of the relationship. The number of close network members with frequent contact is positively related to less depressive symptoms [46]. Especially elderly people rely on members of their immediate family (partner and children) during illness [41]. SHARE provides different questions for these relational dynamics, which we use as controls in our analysis.

For contact frequency, the respondent is asked about the amount of contact with each person in his social support network over the last 12 months. The possible answers are (1) daily, (2) several times a week, (3) about once a week, (4) about every two weeks, (5) about once a month, (6) less than once a month, and (7) never. We recode such that the measure ranges from 0 (never) to 6 (daily). As an overall measure of the amount of network contact of a respondent, we take the average over the answers for each person in his network and call it *contact index*. E.g., if the result is 6 it means that the respondent has daily contact with all persons in his network. If it is less than 6, he must have less than daily contact with at least some member of the network.

Similar measures are constructed for proximity and closeness. The respondent is asked how far the person lives and how close he feels to the person. The categories for closeness are (0) not very close (1) somewhat close (2) very close (3) extremely close; and for proximity (0) more than 500km, (1) 100km to 500km, (2) 25km to 100km, (3) 5km to 25km, (4) 1km to 5km, and (5) less than 1km. The averages over the respective answers for each person in the respondent’s network are the *closeness index* and *proximity index*. Information on the correlation of marriage, the number of children, social network dimensions and well-being measures is given in S3 Table. We observe that the correlations between features of the family status (e.g., married) and the respective network is positive but far from perfect. That is, both people with and without children may indicate that their social support network may predominantly consist of their partner (and similarly for the other network types).

## Results

### Association of marital status and parenthood with well-being and mental health

We present results in an aggregated way to illustrate the relevant patterns, and the robustness of the results with regards to confounding factors. Table 1 shows the associations of the three dimensions of well-being and mental health with family status (number of children, number of resident children, number of grandchildren and marital status) for all respondents (Panel I), male respondents (Panel II), and female respondents (Panel III), over all countries including country fixed effects (further country specific analyses are reported below). The table shows the raw means for each well-being measure conditional on each explanatory variable. Comparing the raw mean values for the well-being measures gives an impression of the effects sizes of each explanatory variable. However, we indicate the significance of each comparison based on regression analyses of the dependent measure on the explanatory variables; the excluded category in the regression analyses is indicated in italics in the table. We show the significance level of the variable and the direction of the association, for the regressions including controls A and B, respectively. For each set of analyses, we also indicate the sample size of the raw means, which varies across analyses because of the variation in the number of respondents in the different modules of the SHARE surveys. We use ordinary least squares for all four measures for its ease of interpretation. Detailed results for each regression are in S4–S6 Tables.

**Table 1:** Regressing well-being and mental health on family status for all countries.

	(I)			(II)			(III)			(IV)		
	Life satisfaction			Quality of life (CASP-12)			Network satisfaction			Lack of depressive symptoms (EURO-D)		
	N	Mean		N	Mean		N	Mean		N	Mean	
**Panel I: All respondents**												
Marriage												
*Not married*	15548	7.14		14902	6.67		15667	8.69		15477	7.50	
Married	36700	7.75	***A,B,+	35610	7.10	***A,B,+	36846	8.90	***A,B,+	36464	8.01	***A,+
Children												
*No*	4746	7.39		4569	6.92		4782	8.53		4731	7.81	
1	9613	7.37		9261	6.84	*A,+	9674	8.82	***A,B,+	9567	7.70	
2	21574	7.64	***A,B,+	20938	7.05	***A,B,+	21676	8.87	***A,B,+	21466	7.97	***A,**B,+
3 or more	16315	7.64	**A,+	15744	6.96	***A,+	16381	8.89	***A,B,+	16177	7.82	
Resident children			*A,***B,-			***A,B,-			*B,-			*B,-
Grandchildren			**B,+			***A,-			***A,B,+			***A,-
**Panel II: Male respondents**												
Marriage												
*Not married*	4576	7.17		4399	6.89		4610	8.42		4556	8.01	
Married	18271	7.78	***A,B,+	17750	7.15	***A,**B,+	18352	8.86	***A,B,+	18149	8.34	***A,+
Children												
*No*	2191	7.37		2114	6.99		2211	8.47		2186	8.15	
1	3885	7.49		3753	6.97	**A,+	3902	8.78	***A,*B,+	3855	8.18	
2	9595	7.73	***A,*B,+	9341	7.18	***A,**B,+	9642	8.81	***A,*B,+	9554	8.37	***A,*B,+
3 or more	7176	7.74	***A,+	6941	7.10	***A,+	7207	8.81	**A,+	7110	8.23	
Resident children						***A.B.-						
Grandchildren			**B,+			***A,-			***A,B,+			**A,-
**Panel III: Female respondents**												
Marriage												
*Not married*	10972	7.12		10503	6.58		11057	8.80		10921	7.29	
Married	18429	7.72	***A,B,+	17860	7.04	***A,B,+	18494	8.94	***A,B,+	18315	7.69	***A,+
Children												
*No*	2555	7.40		2455	6.85		2571	8.58		2545	7.53	
1	5728	7.29		5508	6.75		5772	8.85	***A,B,+	5712	7.39	*A,-
2	11979	7.57	**A,B,+	11597	6.95	**A,*B,+	12034	8.92	***A,B,+	11912	7.65	*B,+
3 or more	9139	7.56		8803	6.85		9174	8.95	***A,B,+	9067	7.50	
Resident children			**A,***B,-			***A,B,-						
Grandchildren						***A,-			***A,B,+			***A,-

*p<0.05

**p<0.01

***p<0.001, +(-) indicates positive (negative) significant effect with the well-being and mental health measure; (I)-(IV) OLS Regression. Controls A: female, age, age^2^, country dummy; Controls B: Controls A, divorced, widowed, education, urban character of residence, employment, self-employment, health status, medication for depressive symptoms, average monthly household income dummy for low, middle, upper middle, and high income (based on country-specific 25^th^, 50^th^ and 75^th^ percentile of the average monthly household income reported in wave 2); Children: Dummy variable for having no children (excluded category), one child, two children, and three or more children. Resident children: Number of children living with their parents. If a respondent has no children then the value is set to 0; Grandchildren: Number of grandchildren, Married: Dummy variable if respondent is married or in registered partnership. Excluded category: Control A: Married but living separated from a spouse, never married, divorced, widowed, Control B: never married since a dummy variable for divorced and widowed is included in Control B. N indicates number of observations in each category for categorical variables.

Overall we observe that marriage is consistently positively correlated with well-being and lack of depressive symptoms, which already provides evidence in favor of hypothesis i) from the introduction. We find that children are positively correlated with well-being and lack of depressive symptoms. However, our analyses show that this overall positive association is due to children after they left home: we find negative effects for the number of resident children. This pattern is consistent with the prediction of hypothesis ii) concerning the effect of non-resident children. Grandchildren correlate positively with life satisfaction and network satisfaction, but negatively with quality of life and lack of depressive symptoms, which gives us a mixed picture on the overall role of grandchildren compared to the prediction in hypothesis ii). While there are some differences in specific correlations, the overall picture is very similar for male and female respondents. Controlling for differences between the countries by conducting separate regressions for each country individually also does not qualitatively change the results (see S7 Table). However, there is clearly heterogeneity in the sense that we observe many null effects next to those effects replicating the overall effects shown in Table 1.

Taken together, the results of Table 1 confirm findings of previous studies [1–4,10,47] for the current large multi country SHARA data set. Focus on the age cohort of people 50 years old and older allows us to identify different associations for children at home, children who left home already, and grandchildren. Given the consistency with previously observed patterns for the direct family status measures, we have a solid foundation for studying the broader role of family in through social networks corresponding to the different family background measures.

### Distribution of social network types

Table 2 presents the means of the network size, the composition measures, and relational dynamic measure of each category. For the distribution of the network size for each network type see S3 Fig. The Partner network is a rather distinct type. It consists only of the partner, i.e. has a size of one, and on average has a contact index close to the maximum. Respondents which are associated with a partner network typically feel extremely or very close with their partner, resulting in the highest closeness index of all network types.

**Table 2:** Network characteristics by social network types.

	All	Partner	Children	Other relatives	Family	Friends	Diverse
	(1)	(C1)	(C2)	(C3)	(C4)	(C5)	(C6)
**Network size**	2.60	1.00	1.93	2.88	3.21	2.95	3.53
**Relationship share**							
Partner	34%	100%	7%	18%	31%	14%	14%
Children	33%	0%	93%	11%	55%	10%	27%
Other Relatives	13%	0%	0%	58%	7%	8%	12%
Friends	16%	0%	0%	11%	7%	64%	12%
Others	6%	0%	0%	2%	1%	3%	34%
**Contact index (0–6)**	5.13	5.99	5.18	4.67	5.21	4.64	4.83
**Closeness index (0–3)**	2.25	2.50	2.35	2.12	2.38	1.99	2.04
**Proximity index (0–5)**	3.99	4.98	3.69	3.51	3.90	3.74	3.86
**# obs.**	50869	9254	6208	6894	13432	8498	6583
**% obs.**^**a**^	100%	18%	12%	14%	26%	17%	13%

Column (1) reports the percentages or means of respondents who have a social network and columns (C1)-(C6) for respondents associated with the respective network type. Network size: number of persons mentioned by the respondent. Relationship categories: Partner, Children, Other relatives, Friends, Other. Contact categories: (0) Never, (1) Less than once a month, (2) About once a month, (3) About every two weeks, (4) About once a week, (5) Several times a week, and (6) Daily. Closeness categories: (0) Not very close (1) Somewhat close (2) Very close (3) Extremely close. Proximity categories: (0) More than 500km, (1) 100km to 500km, (2) 25km to 100km, (3) 5km to 25km, (4) 1km to 5km, and (5) Less than 1km. Contact (closeness, proximity) index: it is defined for each respondent and is the average of the respective measure over all persons in his social support network. Relationship (contact, closeness, proximity) share: it is defined for each category of the measure and each respondent and is the sum of occurrence of each category divided by the size. Values from the same dimension may not add to 100% due to rounding.

^a^1644 respondents report that they do not have a social network (see S4 Table.)

The share of children in the network is highest for the Children network and the Family network. In both networks the second main source of support is the partner, emphasizing the importance of marital status. The Children and Family network differ in terms of the means of the relationship indices and network size, while the average contact and closeness shares are quite similar. The Other Relatives, Friends, and Diverse network types appear to be similar in terms of average contact, closeness and proximity.

**Fig 2:**
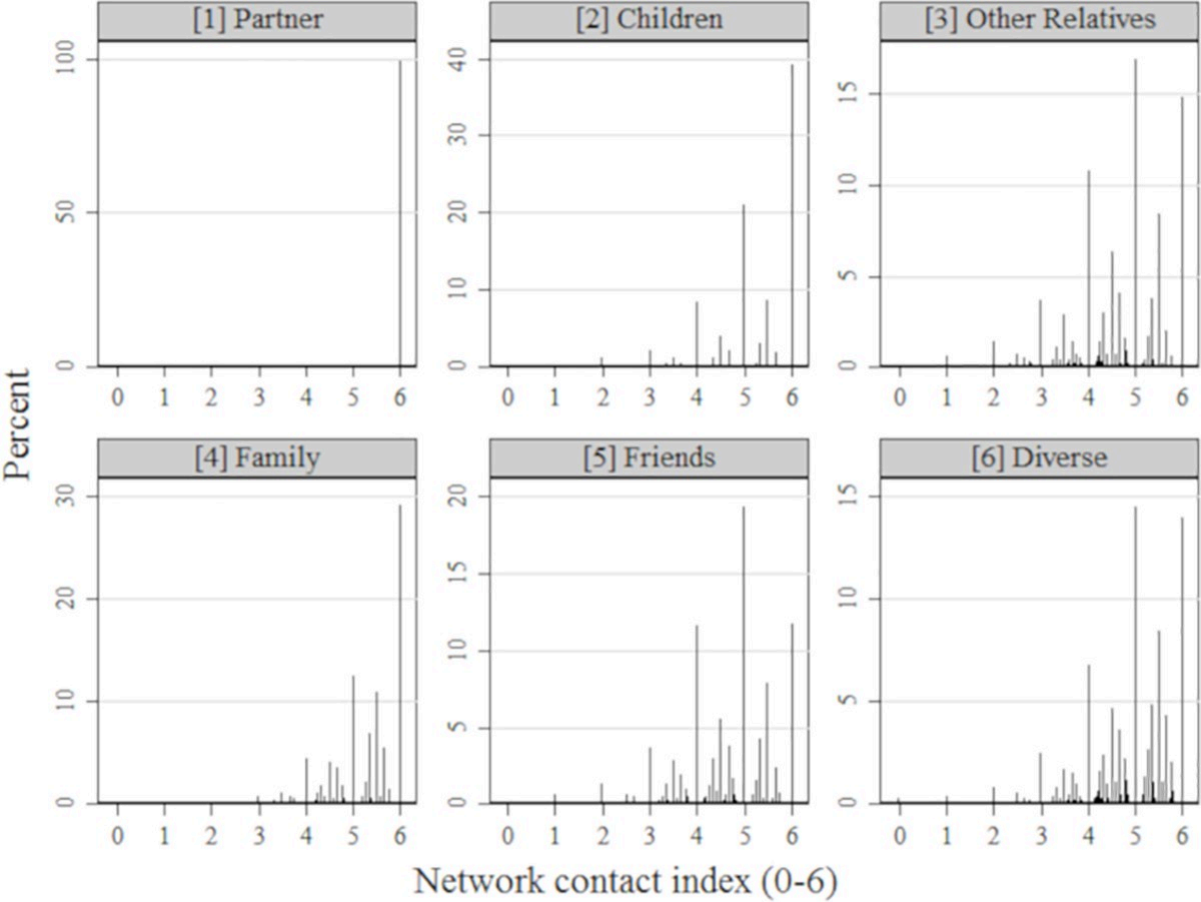
Distribution of the contact index by network type. Each value of the network contact index is represented by a line. The height of each line represents the percentage of the index having the respective value for a network type.

We have shown that network size, as well as contact, proximity, and closeness indices differ across network types on average (see Table 2). Fig 2 presents the full distribution of the contact index for each network type (for the corresponding graphical representations of the proximity and closeness indices, see S4 and S5 Figs). Even network types which are rather similar with respect to the average contact index such as the children and family networks, are rather different with respect to the individual distribution of the network contact index. This means that the actual composition of the different contact categories in the individual social network is different for each network type. Furthermore, the impact of contact, closeness, and proximity could differ, depending on the composition of a given network type: Higher self-reported closeness might, for example, denote a different depth of emotional connection in a Partner, compared to a Friends network. We therefore include the contact, closeness, and proximity indices as individual controls.

Table 3 presents the means of gender, age and family status and the number of observations for the different network types. There are 3% of the respondents who report to have no network. The No network type and Children network type are associated with the lowest share of respondents who are married. Most respondents who have no children are associated with the Other Relatives, the Friends, or the No network type. For the means of further demographic variables see S12 Table.

**Table 3:** Family status by social network types.

Network types	No Network	Partner	Children	Other relatives	Family	Friends	Diverse
	C0	C1	C2	C3	C4	C5	C6
**Female**	49%	35%	72%	61%	56%	61%	63%
**Age at interview**	67	65	71	63	66	64	65
**Marital Status**							
Married/registered partnership	50%	94%	41%	62%	86%	59%	60%
Divorced/living separated	15%	3%	12%	12%	6%	16%	14%
Widowed	23%	1%	45%	12%	7%	15%	19%
**Parenthood**							
Number of children	1.97	2.24	2.55	1.76	2.39	1.82	2.06
Number of resident children	0.29	0.36	0.29	0.33	0.33	0.31	0.35
Number of grandchildren	2.59	2.60	3.89	1.85	2.97	1.91	2.36
Having grandchildren	64%	69%	87%	53%	79%	57%	66%
**% obs.**	3%	18%	12%	13%	26%	16%	13%

Columns (C0)-(C6) report the percentages or means of respondents associated with the respective network type. There are in total 52513 observations. Values from the same dimension may not add to 100% due to rounding.

### Association of social networks with well-being and mental health

We can now turn to the relationship between network characteristics and well-being and mental health measures. Table 4 compares well-being and mental health for respondents who have a social support network, and those who have no network at all. We use the different network types as explanatory variable, and include network size, family status variables (as in Table 1), and socioeconomic variables as controls. We do not control for relational dynamics, because these measures are not defined for those who have no social support network. For the detailed regression results see S8–S10 Tables and for the raw means conditional on network size see S11 Table.

**Table 4:** Regressing well-being and mental health on social support network types; controlling for network size and family status for all countries.

	(I)			(II)			(III)			(IV)		
Dependent Variable	Life satisfaction			Quality of life (CASP-12)			Network satisfaction			Lack of depressive symptoms (EURO-D)		
	N	Mean		N	Mean		N	Mean		N	Mean	
**Panel I: All respondents**												
Network type												
*No network*	1624	6.93		1555	6.48		1644	6.49		1615	7.59	
Partner	9209	7.71	***A,B,+	8944	7.05	***A,B,+	9254	8.98	***A,B,+	9145	8.21	***A,B,+
Children	6157	7.37	***A,B,+	5909	6.52		6208	9.10	***A,B,+	6116	7.49	***B,+
Other Relatives	6856	7.44	***A,B,+	6622	7.06	*B,+	6894	8.77	***A,B,+	6805	7.76	
Family	13381	7.77	***A,B,+	12989	7.05	*B,+	13432	9.05	***A,B,+	13316	7.98	*A.***B,+
Friends	8468	7.52	***A,B,+	8200	7.13	**B,+	8498	8.73	***A,B,+	8423	7.82	
Diverse	6553	7.5	***B,+	6293	6.92		6583	8.74	***A,B,+	6521	7.68	*A,-
Network size			***A,B,+			***A,B,+			***A,B,+			***A,**B,+
**Panel I: Male respondents**												
Network type												
*No network*	829	6.98		790	6.67		841	6.60		823	7.99	
Partner	5996	7.70	***A,B,+	5820	7.06	*A,B,+	6026	9.03	***A,B,+	5955	8.36	**A,B,+
Children	1755	7.77	***A,B,+	1695	7.03		1765	9.06	***A,B,+	1745	8.17	*B,+
Other Relatives	2656	7.47	***A,B,+	2576	7.15		2671	8.65	***A,B,+	2634	8.18	
Family	5895	7.77	***A,B,+	5732	7.07		5922	9.00	***A,B,+	5870	8.31	**B,+
Friends	3310	7.64	***A,B,+	3218	7.27	*A,B,+	3322	8.58	***A,B,+	3286	8.25	
Diverse	2406	7.65	**A,B,+	2318	7.16		2415	8.54	***A,B,+	2392	8.23	
Network size			***A,B,+			***A,B,+			***A,B,+			***A,+
**Panel II: Female respondents**												
Network type												
*No network*	795	6.88		765	6.29		803	6.38		792	7.18	
Partner	3213	7.71	***A,B,+	3124	7.03	***A,B,+	3228	8.90	***A,B,+	3190	7.93	***A,B,+
Children	4402	7.21	**B,+	4214	6.32		4443	9.12	***A,B,+	4371	7.22	**B,+
Other Relatives	4200	7.43	**B,+	4046	6.99		4223	8.84	***A,B,+	4171	7.49	
Family	7486	7.77	**A,***B,+	7257	7.04	*B,+	7510	9.10	***A,B,+	7446	7.72	***B,+
Friends	5158	7.45	*B,+	4982	7.05	*B,+	5176	8.82	***A,B,+	5137	7.55	
Diverse	4147	7.41	**B,+	3975	6.79		4168	8.86	***A,B,+	4129	7.36	
Network size			***A,B,+			***A,B,+			***A,B,+			***A,*B,+

*p<0.05

**p<0.01

***p<0.001, +(-) indicates positive (negative) significant effect with the well-being measure; (I)-(IV) OLS Regression. Controls A: female, age, age^2^, country dummy; Controls B: Controls A, divorced, widowed, education, household size, urban character of residence, retired, self-employment, health status, average monthly household income dummy for low, middle, upper middle, and high income (based on country-specific 25^th^, 50^th^ and 75^th^ percentile of the average monthly household income reported in wave 2). Children, resident children, grandchildren and married included in both A and B. Network types: the excluded category is having no network. N indicates number of observations in each category for categorical variables.

In accordance with hypothesis iii), all network types relate positively to measures of well-being, for both males and females, even after controlling for family structure. The effect is consistently observed for Life satisfaction and Network satisfaction. For CASP-12 the effect is observed for Partner and Friends network types for male respondents and for Partner, Family and Friends network types for female respondents. Interestingly, the positive relationship with Children network and Lack of depressive symptoms mostly emerges only after inclusion of the full set of controls. Network size is positively related to all measures of well-being [37]. The results obtained in this network analysis support the broader relevance of family through the resulting networks for well-being and mental health as postulated in hypotheses i) and ii).

We next compare the different network types with each other, accounting for variation of the relational indices across the different network types. Table 5 shows the results confined to respondents who indicated the presence of some social support network. For the detailed regression results see S12–S14 Tables. The excluded category of the network type is the Partner network, which had consistently strong and significant associations in Table 4, and is taken as a benchmark here.

**Table 5:** Regressing well-being and mental health on social support network types (only for respondents with a social network); controlling for network size, relational dynamic measures, and family status for all countries.

	(I)	(II)	(III)	(IV)
Dependent Variable	Life satisfaction	Quality of life (CASP-12)	Network satisfaction	Lack of depressive symptoms (EURO-D)
**Panel I: all respondents**				
Network type^a^				
Children	***A,B,-	***A,B,-	***A,B,+	***A,*B,-
Other Relatives	***A,-		***A,B,+	***A,B,-
Family	***A,-	***A,-	***A,B,+	***A,-
Friends		***B,+	***A,B,+	***A,B,-
Diverse	***A,-	***A,-		***A,B,-
Network size	***A,B,+	***A,B,+	***A,B,+	***A,B,+
Contact Index	***A,B,+	***A,B,+	***A,B,+	**A,B,+
Closeness Index	***A,B,+	***A,B,+	***A,B,+	***A,B,+
Proximity Index	***A,-	******A,B,-	***A,B,-	**A,-
**Panel II: male respondents**^**b**^				
Network type ^a^				
Children	*A,-	*A,-	***A,B,+	*A,-
Other Relatives		*B,+		*A,-
Family		*A,-		
Friends		***A,B,+		
Diverse				*A,-
Network size	***A,B,+	***A,B,+	***A,B,+	***A,*B,+
Contact Index	**A,B,+	**A,***B,+	***A,B,+	
Closeness Index	***A,B,+	***A,B,+	***A,B,+	***A,B,+
Proximity Index	**A,-	***A,-	***A,B,-	
**Panel III: female respondents**^**b**^				
Network type ^a^				
Children	***A,B,-	***A,B,-	***A,B,+	***A,B,-
Other Relatives	***A,*B,-	***A,-	***A,B,+	***A,B,-
Family	***A,-	***A,**B,-	***A,B,+	***A,**B,-
Friends	***A,*B,-		***A,B,+	***A,B,-
Diverse	***A,*B,-	***A,*B,-	***A,B,+	***A,B,-
Network size	***A,B,+	***A,B,+	***A,B,+	***A,**B,+
Contact Index	***A,B,+	***A,B,+	***A,B,+	*A,B,+
Closeness Index	***A,B,+	***A,B,+	***A,B,+	***A,B,+
Proximity Index	**A,-	***A,**B,-	***A,B,-	**A,-

*p<0.05

**p<0.01

***p<0.001, +(-) indicates positive (negative) significant effect with the well-being measure; (I)-(IV) OLS Regression. Controls A: female, age, age^2^, country dummy; Controls B: Controls A, divorced, widowed, education, household size, urban character of residence, retired, self-employment, health status, average monthly household income dummy for low, middle, upper middle, and high income (based on country-specific 25^th^, 50^th^ and 75^th^ percentile of the average monthly household income reported in wave 2). Children, resident children, grandchildren and married included in both A and B.

^a^Partner network is the excluded category

^b^Control female is excluded

We find that for Life satisfaction, CASP-12, and Lack of depressive symptoms, the more diverse networks have typically weaker associations than the Partner network, with the exception of the Friends network for CASP-12. In contrast, Network satisfaction is consistently higher for all other networks, except for the Diverse network. Note, that this emerges despite controlling for network size, contact, closeness and proximity index. Fiori et al. [42] pointed out that support quality is an important factor for depressive symptoms. We find consistently that the closeness and contact measure is positively correlated with mental health and well-being. However, we observe a negative relationship of mere proximity with well-being and mental health. While for the associations with family status no relevant gender differences were observed, we observe that associations with network types differ for male and female respondents. For male respondents in most cases the effects of the network types are not significantly different from the Partner network. For the females, the above discussed associations show up significantly.

## Discussion

Reflecting the results presented in the previous section in terms of our three research hypotheses, the following implications for the well-being and mental health of people aged 50 and above emerge: i) There appears to be a strong positive association of being married/having a partner as part of a social network. ii) Non-residential children also relate positively to well-being and mental health. On the other hand, the effect of grandchildren in general appears to be mixed. While they may be associated with higher life and network satisfaction, the same does not appear to hold for depressive symptoms and perceived quality of life. iii) We find clear evidence of positive relationhips of all types of social networks with our measures of well-being, over and beyond the respective underlying family status indicators. Hence, a simple focus on family status measures, not accounting for the resulting network structures, misses important aspects of the relationship of family and well-being and mental health.

In contrast to negative associations reported in many studies (for an overview see Hansen [7], or the discussion in Nelson et al. [48] and Herbst and Ifcher [9]), we find that children are indeed positively correlated with well-being and lack of depressive symptoms, when controlling for residential status (resident children are negatively associated with well-being). This result is consistent with age-dependence in the correlation of children with well-being [1,10] and mental health [4,47]. The results suggest that the finding of a negative link between children and well-being and mental health may not generalize to older people whose children have often left home already. As stress associated with balancing the competing demands of childcare, work and personal life decreases, once people get older and their children leave house, the importance of children as caregivers and social contacts might prevail. The mixed effect of grandchildren is more difficult to explain. Potentially, there are positive effects of having grandchildren in terms of social support that might coincide with negative aspects, such as having to care for these grandchildren [49,50]. As the SHARE data set only provides us with rudimentary information about grandchildren (there is for example no information about the residency of them), we cannot shed more light on the relation between grandchildren and well-being, as well as mental health.

We observe that all types of networks have positive associations with our dependent measures. Network characteristics such as size, closeness, contact frequency and proximity are also relevant indicators of well-being and mental health. For male respondents in most cases the effects of the network types are not significantly different from the Partner network. For female respondents, on the other hand, we observe more cases where associations of well-being and mental health with the Partner network type are significantly different from those for the other network types. Overall we find that especially the Partner network is consistently positively correlated with well-being and mental health, despite the small network size of 1. This is in contrast to Litwin and Stoeckel [16,51], who found that the Spouse network is not significantly related to well-being. However, importantly, because we control for network size separately, positive associations with size are captured by this variable. A remarkable feature of the findings in Table 4 is that network characteristics are positively associated with well-being and mental health even after controlling for the above-shown associations with family status indicators. That is, a healthy partner network captures more than just being married, as do other types of networks. This fits previous results, suggesting that it is not being married per se, but being satisfied with the relationship that is associated with less depressive symptoms [47,52]. Kim and McKenry also report both, a positive relationship of well-being with being married, and an additional role for the perceived quality of the marriage on top of that [53]. Our research extends these previous studies, by demonstrating the role of both the presence of a partner and the associated network, where the partner actively provides social support. As the size of a social network seems to be an important driver of subjective well-being [54], this could indicate that a small partner network can offset the lack of a larger social network. Unfortunately, a limitation of our present study is that besides general network satisfaction, the SHARE data set has no more fine-grained questions for the quality of marriages/partnerships.

Taken together, our results suggest that social networks may be important for well-being and mental health in old age. Spouses, partners and children are often the basis of long-lasting social networks, which can provide social support to elderly people. However, different forms of network may have similar effects, as our data especially for male respondents suggests. As discussed above, this might derive from a level of trust and reciprocity implicit in all forms of networks. A remaining limitation of our study is of course that the results are correlational in nature. Further studies, comparing for example well-being and mental health before and after the formation of partnerships or social networks in longitudinal data are needed to establish which factors cause the positive effects found here and in the literature. Furthermore, research suggests that there is an important link between social support obtained from social networks and subjective well-being [54,55]. Subjective well-being is commonly measured with questions concerning life satisfaction, positive affect (experiencing positive emotions), and negative affect (experiencing negative emotions). As the SHARE data set only measures life satisfaction, we cannot draw a complete picture of the effects of family status and social networks on broader measures of subjective well-being.

Networks may exert an influence on the person’s life beyond the mere role of the corresponding family status, for example by moderating influences of the environment on well-being. The direct association of family status with well-being and mental health may not capture such effects. Importantly, the current insights need to guide further research, with the next step being the assessment of the causal direction of the reported associations. This will allow moving towards making recommendations for public policy to maintain the well-being and mental health of the elderly through social networks.

## Supporting information

S1 Fig Age distribution. Percent of male (female) respondents for each age.
(TIF)

S2 Fig Average well-being and mental health measure, by gender. Average well-being and mental health measure for all ages from 50 to 90 years for male and female respondents. Male: black lines, Female: grey lines. After age 91 the number of available observations drops to less than 50.
(TIF)

S3 Fig Network size by network type. The size of a bar reflects the share of respondents in a network type having a network of size 0 to 7.
(TIF)

S4 Fig Distribution of the proximity index by network type. Each value of the network contact index is represented by a line. The height of each line represents the percentage of the index having the respective value for a network type.
(TIF)

S5 Fig Distribution of the closeness index by network type. Each value of the network contact index is represented by a line. The height of each line represents the percentage of the index having the respective value for a network type.
(TIF)

S1 Table Number of observations and unfolding income brackets per country. (DOCX)

S2 Table Summary statistics of demographic variables. (DOCX)

S3 Table Correlation of family status, social network characteristics, well-being and mental health. (DOCX)

S4 Table Regressing well-being and mental health on family status for all countries, all respondents. (DOCX)

S5 Table Regressing well-being and mental health on family status for all countries, male respondents. (DOCX)

S6 Table Regressing well-being and mental health on family status for all countries, female respondents. (DOCX)

S7 Table Regressing well-being and mental health on family status for each country. (DOCX)

S8 Table Regressing well-being and mental health on network types controlling for network size and family status for all countries, all respondents. (DOCX)

S9 Table Regressing well-being and mental health on network types controlling for network size and family status for all countries, male respondents. (DOCX)

S10 Table Regressing well-being and mental health on network types controlling for network size and family status for all countries, female respondents. (DOCX)

S11 Table Well-being and mental health measures conditional on network size over all countries. (DOCX)

S12 Table Regressing well-being and mental health on network types controlling for network size, relational dynamics and family status for all countries, all respondents with social support network. (DOCX)

S13 Table Regressing well-being and mental health on network types controlling for network size, relational dynamics and family status for all countries, male respondents with social support network. (DOCX)

S14 Table Regressing well-being and mental health on network types controlling for network size, relational dynamics and family status for all countries, female respondents with social support network. (DOCX)
