# Supplementary figures and images for: Marriage, parenthood and social network: Subjective well-being and mental health in old age
Source: PLoS One. 2019 Jul 24;14(7):e0218704. doi: 10.1371/journal.pone.0218704 (PMC6656342; doi:10.1371/journal.pone.0218704)

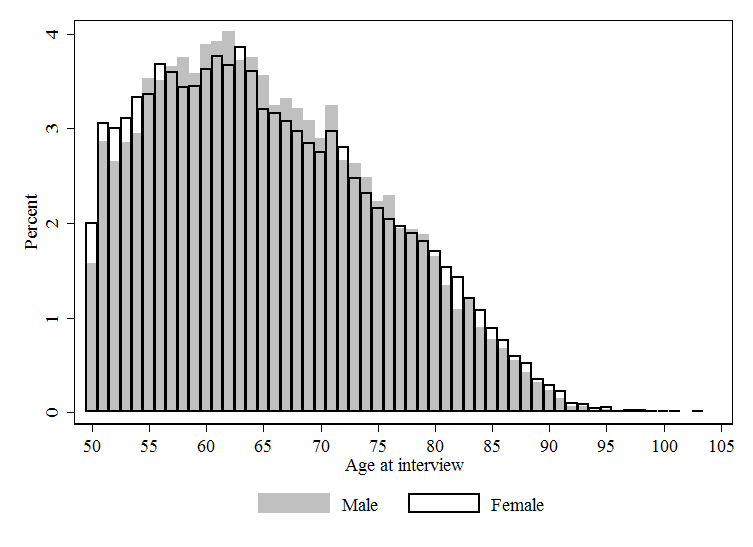

Supplement: S1 Fig — Percent of male (female) respondents for each age. (TIF) [file pone.0218704.s001.tif]

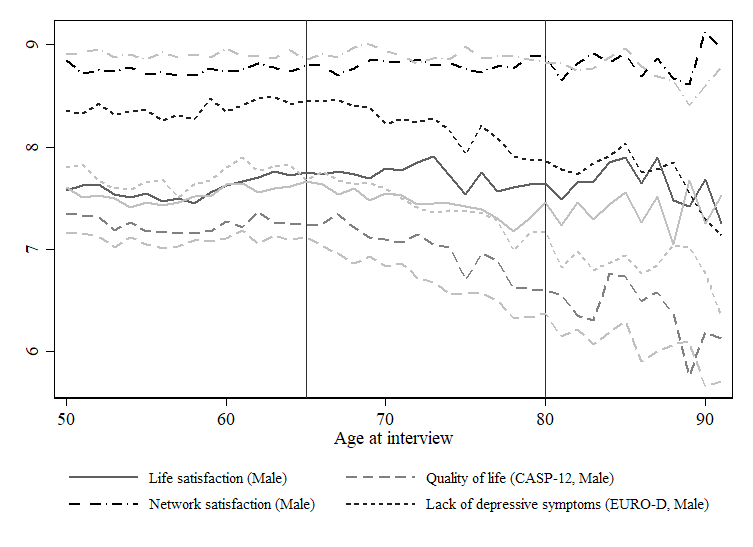

Supplement: S2 Fig — Average well-being and mental health measure for all ages from 50 to 90 years for male and female respondents. Male: black lines, Female: grey lines. After age 91 the number of available observations drops to less than 50. (TIF) [file pone.0218704.s002.tif]

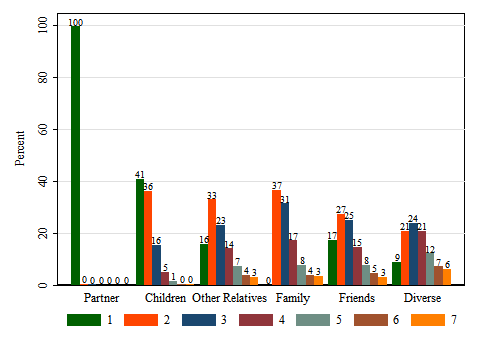

Supplement: S3 Fig — The size of a bar reflects the share of respondents in a network type having a network of size 0 to 7. (TIF) [file pone.0218704.s003.tif]

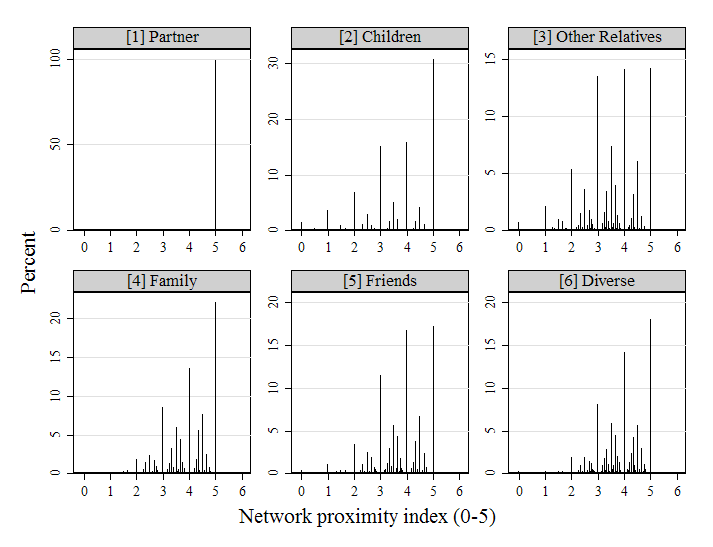

Supplement: S4 Fig — Each value of the network contact index is represented by a line. The height of each line represents the percentage of the index having the respective value for a network type. (TIF) [file pone.0218704.s004.tif]

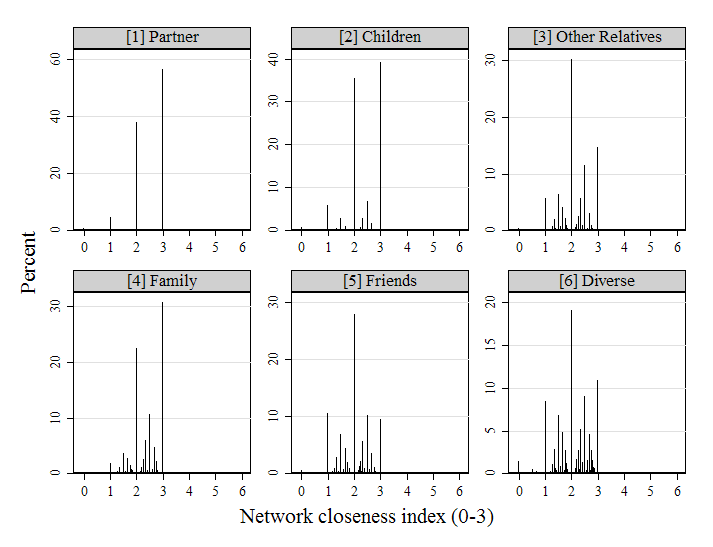

Supplement: S5 Fig — Each value of the network contact index is represented by a line. The height of each line represents the percentage of the index having the respective value for a network type. (TIF) [file pone.0218704.s005.tif]
