# Supplementary material for: Marriage, parenthood and social network: Subjective well-being and mental health in old age
Source: PLoS One. 2019 Jul 24;14(7):e0218704. doi: 10.1371/journal.pone.0218704 (PMC6656342; doi:10.1371/journal.pone.0218704)
Supplement: S1 Table — (DOCX) [file pone.0218704.s006.docx]

**S1 Table. Number of observations and unfolding income brackets per country**

|  |  |  | Unfolding brackets in € | | | Percent of household income | | | |
| --- | --- | --- | --- | --- | --- | --- | --- | --- | --- |
| Country | # obs. | Percent | Low | Middle | High | [0,Low) | [Low,Middle) | [Middle,High) | [High,) |
| Austria | 4808 | 9% | 1500 | 2000 | 3000 | 30% | 17% | 24% | 29% |
| Belgium | 4844 | 9% | 1500 | 2000 | 3000 | 28% | 20% | 24% | 28% |
| Czech Republic | 4898 | 9% | 1629 | 2037 | 2851 | 84% | 5% | 2% | 9% |
| Denmark | 2074 | 4% | 1342 | 2013 | 2684 | 9% | 15% | 16% | 60% |
| Estonia | 6330 | 12% | 192 | 256 | 320 | 3% | 4% | 12% | 81% |
| France | 5111 | 10% | 1500 | 2000 | 3000 | 33% | 17% | 23% | 28% |
| Germany | 1429 | 3% | 1500 | 2000 | 3000 | 27% | 21% | 25% | 27% |
| Hungary | 2901 | 6% | 1444 | 2166 | 2888 | 91% | 2% | 1% | 6% |
| Italy | 3317 | 6% | 1500 | 2000 | 3000 | 45% | 18% | 18% | 19% |
| Netherlands | 2498 | 5% | 1500 | 2000 | 3000 | 19% | 22% | 29% | 30% |
| Poland | 1486 | 3% | 1418 | 1890 | 2363 | 82% | 1% | 1% | 16% |
| Portugal | 1837 | 3% | 1500 | 2000 | 3000 | 64% | 10% | 7% | 19% |
| Slovenia | 2600 | 5% | 1500 | 2000 | 3000 | 69% | 12% | 7% | 11% |
| Spain | 3081 | 6% | 1500 | 2000 | 3000 | 57% | 12% | 10% | 21% |
| Sweden | 1816 | 3% | 1108 | 2215 | 3323 | 7% | 30% | 30% | 32% |
| Switzerland | 3483 | 7% | 1620 | 2025 | 2430 | 10% | 5% | 6% | 79% |
| Total | 52513 |  |  |  |  |  |  |  |  |

Low, Middle, and High are the 25^th^, 50^th^, and 75^th^ percentile of the reported household incomes from SHARE wave 2 (Field time 2006-2007). If a respondent refuses to state the amount of the overall income, after tax that the entire household had in an average month of the last year the interviewer asks the following questions, starting with the lowest threshold: Do you earn a) more than this amount, b) less than this amount or c) approximately this amount. The boundaries of the intervals are the respective country-specific 25^th^, 50^th^, and 75^th^ percentile of the reported household incomes from SHARE wave 2 which are used as unfolding brackets in SHARE wave 4.
