# Supplementary material for: Marriage, parenthood and social network: Subjective well-being and mental health in old age
Source: PLoS One. 2019 Jul 24;14(7):e0218704. doi: 10.1371/journal.pone.0218704 (PMC6656342; doi:10.1371/journal.pone.0218704)
Supplement: S2 Table — (DOCX) [file pone.0218704.s007.docx]

**S2 Table. Summary statistics of demographic variables**

|  | All (1) | Male (2) | Female (3) | All (4) |
| --- | --- | --- | --- | --- |
| **Female** | 56% | 0% | 100% | 52513 |
| **Age at interview** | 66 | 66 | 66 | 52513 |
| **Parenthood** |  |  |  |  |
| Number of children | 2.15 | 2.16 | 2.15 | 52513 |
| Having children | 91% | 90% | 91% | 52513 |
| No children | 9% | 10% | 9% | 52513 |
| One child | 18% | 17% | 20% | 52513 |
| Two children | 41% | 42% | 41% | 52513 |
| Three or more children | 31% | 31% | 31% | 52513 |
| Number of resident children^a^ | 0.33 | 0.36 | 0.31 | 52513 |
| Having resident children | 24% | 24% | 23% | 52513 |
| No resident child^b^ | 74% | 73% | 75% | 47731 |
| All children are resident^b^ | 9% | 10% | 8% | 47731 |
| Number of grandchildren | 2.61 | 2.43 | 2.74 | 52513 |
| Having grandchildren | 69% | 66% | 71% | 52513 |
| **Marital Status** |  |  |  |  |
| Married/registered partnership | 70% | 80% | 63% | 52513 |
| Divorced/living separated | 10% | 8% | 11% | 52513 |
| Widowed | 14% | 6% | 21% | 52513 |
| **Housing** |  |  |  |  |
| Household size | 2.16 | 2.28 | 2.06 | 52513 |
| Big city | 14% | 14% | 15% | 50879 |
| Suburbs of big city | 10% | 11% | 10% | 50879 |
| Large town | 16% | 16% | 17% | 50879 |
| Small town | 24% | 24% | 24% | 50879 |
| Rural area/village | 35% | 36% | 34% | 50879 |
| **Average monthly household income** |  |  |  |  |
| Low income | 38% | 35% | 41% | 48736 |
| Middle income | 13% | 13% | 12% | 48736 |
| Upper middle income | 15% | 15% | 15% | 48736 |
| High income | 34% | 37% | 32% | 48736 |
| **Employed** | 25% | 26% | 23% | 52466 |
| **Self-employed** | 6% | 8% | 4% | 52466 |
| **Education** |  |  |  |  |
| None | 3% | 2% | 3% | 52179 |
| Primary school | 19% | 17% | 21% | 52179 |
| Lower secondary school | 19% | 18% | 21% | 52179 |
| Upper secondary school | 34% | 37% | 32% | 52179 |
| Post-secondary non-tertiary education | 5% | 5% | 5% | 52179 |
| First stage tertiary education | 19% | 21% | 18% | 52179 |
| Second stage tertiary education | 1% | 1% | 1% | 52179 |
| **Health status** |  |  |  |  |
| Poor | 12% | 11% | 13% | 52500 |
| Fair | 30% | 28% | 31% | 52500 |
| Good | 35% | 36% | 35% | 52500 |
| Very good | 16% | 18% | 15% | 52500 |
| Excellent | 7% | 7% | 6% | 52500 |
| Medication for depressive symptoms | 13% | 8% | 17% | 52461 |

Columns (1)-(3) report the percentages or means of all respondents, and by gender. Column (4) shows the total number of observations. SHARE is using the international classification of education ISCED-97 with which education can be classified according to internationally agreed set of definitions and concepts (UNESCO 1997). Medication for depressive symptoms is equal to one if the respondent takes drugs for sleeping problems, anxiety or depression. Values from the same dimension may not add to 100% due to rounding.

^b^The number of resident children is inferred from matching the age/gender information from the persons living with the family (Coverscreen module) and the age/gender information from the children of the respondents (Children module).

^a^Percentage conditional on having a child.

UNESCO. 1997. “International Standard Classification of Education ISCED 1997.”
