# Supplementary material for: Marriage, parenthood and social network: Subjective well-being and mental health in old age
Source: PLoS One. 2019 Jul 24;14(7):e0218704. doi: 10.1371/journal.pone.0218704 (PMC6656342; doi:10.1371/journal.pone.0218704)
Supplement: S3 Table — (DOCX) [file pone.0218704.s008.docx]

**S3 Table. Correlation of family status, social network characteristics, well-being and mental health**

Correlations with a p-value smaller than 0.05 are shown. Network size: number of persons mentioned by the respondent. Relationship categories: Partner, Children, Other relatives, Friends, Other. Contact categories: (0) Never, (1) Less than once a month, (2) About once a month, (3) About every two weeks, (4) About once a week, (5) Several times a week, and (6) Daily. Closeness categories: (1) Not very close (2) Somewhat close (3) Very close (4) Extremely close. Proximity categories: (0) More than 500km, (1) 100km to 500km, (2) 25km to 100km, (3) 5km to 25km, (4) 1km to 5km, and (5) Less than 1km. Contact (closeness, proximity) index: it is defined for each respondent and is the average of the respective measure over all persons in his social support network. Relationship share: it is defined for each category of the measure and each respondent and is the sum of occurrence of each category divided by the size.
