# Supplementary material for: Marriage, parenthood and social network: Subjective well-being and mental health in old age
Source: PLoS One. 2019 Jul 24;14(7):e0218704. doi: 10.1371/journal.pone.0218704 (PMC6656342; doi:10.1371/journal.pone.0218704)
Supplement: S4 Table — (DOCX) [file pone.0218704.s009.docx]

**S4 Table. Regressing well-being and mental health on family status for all countries, all respondents**

|  | Life satisfaction | | Quality of life (CASP-12) | | Network satisfaction | | Lack of depressive symptoms (EURO-D) | |
| --- | --- | --- | --- | --- | --- | --- | --- | --- |
|  | A | B | A | B | A | B | A | B |
| Married/registered partnership | 0.56*** | 0.44*** | 0.30*** | 0.20*** | 0.21*** | 0.26*** | 0.28*** | 0.039 |
|  | (0.000) | (0.000) | (0.000) | (0.000) | (0.000) | (0.000) | (0.000) | (0.321) |
| [1] Having 1 child | 0.0046 | -0.025 | 0.066* | 0.028 | 0.22*** | 0.17*** | -0.043 | -0.015 |
|  | (0.891) | (0.466) | (0.035) | (0.362) | (0.000) | (0.000) | (0.206) | (0.651) |
| [2] Having 2 children | 0.18*** | 0.11*** | 0.19*** | 0.11*** | 0.24*** | 0.19*** | 0.11*** | 0.095** |
|  | (0.000) | (0.001) | (0.000) | (0.000) | (0.000) | (0.000) | (0.001) | (0.003) |
| [3] Having 3 or more children | 0.11** | 0.057 | 0.13*** | 0.051 | 0.23*** | 0.17*** | 0.021 | 0.019 |
|  | (0.001) | (0.113) | (0.000) | (0.123) | (0.000) | (0.000) | (0.555) | (0.588) |
| Number of resident children | -0.031* | -0.054*** | -0.097*** | -0.12*** | -0.016 | -0.023* | -0.020 | -0.027* |
|  | (0.019) | (0.000) | (0.000) | (0.000) | (0.130) | (0.047) | (0.152) | (0.033) |
| Number of grandchildren | -0.0050 | 0.010** | -0.022*** | -0.00015 | 0.015*** | 0.018*** | -0.020*** | -0.0022 |
|  | (0.173) | (0.003) | (0.000) | (0.961) | (0.000) | (0.000) | (0.000) | (0.521) |
| **Controls** |  |  |  |  |  |  |  |  |
| Female | -0.030 | 0.053*** | -0.15*** | -0.041** | 0.14*** | 0.15*** | -0.65*** | -0.51*** |
|  | (0.056) | (0.001) | (0.000) | (0.003) | (0.000) | (0.000) | (0.000) | (0.000) |
| Age at interview | 0.023* | 0.043*** | 0.12*** | 0.13*** | -0.013 | -0.0099 | 0.12*** | 0.11*** |
|  | (0.030) | (0.000) | (0.000) | (0.000) | (0.136) | (0.307) | (0.000) | (0.000) |
| Age at interview, squared | -0.00014 | -0.00015 | -0.0010*** | -0.00098*** | 0.000088 | 0.000078 | -0.00100*** | -0.00077*** |
|  | (0.068) | (0.062) | (0.000) | (0.000) | (0.166) | (0.265) | (0.000) | (0.000) |
| sh_country==[2]BEL | -0.53*** | -0.45*** | -0.78*** | -0.65*** | -0.65*** | -0.65*** | -0.49*** | -0.29*** |
|  | (0.000) | (0.000) | (0.000) | (0.000) | (0.000) | (0.000) | (0.000) | (0.000) |
| sh_country==[3]CHE | 0.094** | -0.12*** | 0.26*** | 0.029 | -0.36*** | -0.39*** | -0.011 | -0.20*** |
|  | (0.005) | (0.000) | (0.000) | (0.375) | (0.000) | (0.000) | (0.758) | (0.000) |
| sh_country==[4]CZE | -0.99*** | -0.62*** | -1.44*** | -1.00*** | -0.42*** | -0.37*** | -0.24*** | 0.13*** |
|  | (0.000) | (0.000) | (0.000) | (0.000) | (0.000) | (0.000) | (0.000) | (0.000) |
| sh_country==[5]DEU | -0.56*** | -0.46*** | -0.27*** | -0.15*** | -0.43*** | -0.42*** | -0.26*** | -0.14** |
|  | (0.000) | (0.000) | (0.000) | (0.001) | (0.000) | (0.000) | (0.000) | (0.002) |
| sh_country==[6]DNK | 0.27*** | 0.021 | 0.24*** | -0.033 | 0.061 | -0.00070 | 0.15*** | -0.031 |
|  | (0.000) | (0.589) | (0.000) | (0.343) | (0.052) | (0.983) | (0.000) | (0.437) |
| sh_country==[7]ESP | -0.76*** | -0.37*** | -1.10*** | -0.54*** | -0.31*** | -0.25*** | -0.69*** | -0.21*** |
|  | (0.000) | (0.000) | (0.000) | (0.000) | (0.000) | (0.000) | (0.000) | (0.000) |
| sh_country==[8]EST | -1.58*** | -1.24*** | -1.23*** | -0.80*** | -0.45*** | -0.38*** | -0.95*** | -0.44*** |
|  | (0.000) | (0.000) | (0.000) | (0.000) | (0.000) | (0.000) | (0.000) | (0.000) |
| sh_country==[9]FRA | -1.01*** | -0.84*** | -0.54*** | -0.29*** | -0.58*** | -0.56*** | -0.68*** | -0.40*** |
|  | (0.000) | (0.000) | (0.000) | (0.000) | (0.000) | (0.000) | (0.000) | (0.000) |
| sh_country==[10]HUN | -1.60*** | -1.03*** | -1.38*** | -0.68*** | -0.17*** | -0.10* | -0.95*** | -0.35*** |
|  | (0.000) | (0.000) | (0.000) | (0.000) | (0.000) | (0.012) | (0.000) | (0.000) |
| sh_country==[11]ITA | -0.74*** | -0.51*** | -1.68*** | -1.37*** | -0.39*** | -0.36*** | -0.62*** | -0.37*** |
|  | (0.000) | (0.000) | (0.000) | (0.000) | (0.000) | (0.000) | (0.000) | (0.000) |
| sh_country==[12]NLD | -0.29*** | -0.35*** | 0.24*** | 0.19*** | -0.62*** | -0.65*** | 0.076 | 0.069 |
|  | (0.000) | (0.000) | (0.000) | (0.000) | (0.000) | (0.000) | (0.054) | (0.069) |
| sh_country==[13]POL | -0.93*** | -0.36*** | -1.20*** | -0.53*** | -0.25*** | -0.17*** | -1.03*** | -0.45*** |
|  | (0.000) | (0.000) | (0.000) | (0.000) | (0.000) | (0.001) | (0.000) | (0.000) |
| sh_country==[14]PRT | -1.31*** | -0.64*** | -2.16*** | -1.32*** | -0.095* | 0.098* | -1.17*** | -0.35*** |
|  | (0.000) | (0.000) | (0.000) | (0.000) | (0.011) | (0.020) | (0.000) | (0.000) |
| sh_country==[15]SVN | -0.89*** | -0.60*** | -0.17*** | 0.22*** | -0.44*** | -0.39*** | -0.38*** | -0.12** |
|  | (0.000) | (0.000) | (0.000) | (0.000) | (0.000) | (0.000) | (0.000) | (0.004) |
| sh_country==[16]SWE | 0.031 | -0.10* | -0.20*** | -0.31*** | -0.086* | -0.13*** | 0.024 | -0.017 |
|  | (0.456) | (0.015) | (0.000) | (0.000) | (0.017) | (0.000) | (0.574) | (0.682) |
| Divorced/living separated |  | -0.10* |  | -0.050 |  | -0.0082 |  | -0.13** |
|  |  | (0.034) |  | (0.225) |  | (0.849) |  | (0.003) |
| Widowed |  | 0.067 |  | 0.10** |  | 0.13** |  | -0.15*** |
|  |  | (0.151) |  | (0.010) |  | (0.002) |  | (0.001) |
| [1] Suburbs of big city |  | 0.0080 |  | 0.017 |  | 0.033 |  | -0.079** |
|  |  | (0.793) |  | (0.528) |  | (0.219) |  | (0.010) |
| [2] Large town |  | 0.038 |  | 0.019 |  | 0.077** |  | -0.067* |
|  |  | (0.183) |  | (0.456) |  | (0.002) |  | (0.016) |
| [3] Small town |  | 0.11*** |  | 0.066** |  | 0.092*** |  | 0.024 |
|  |  | (0.000) |  | (0.005) |  | (0.000) |  | (0.340) |
| [4] Rural area/village |  | 0.059* |  | 0.045* |  | 0.034 |  | 0.0038 |
|  |  | (0.022) |  | (0.048) |  | (0.116) |  | (0.876) |
| Employment, current job |  | 0.18*** |  | 0.17*** |  | 0.026 |  | 0.093*** |
|  |  | (0.000) |  | (0.000) |  | (0.171) |  | (0.000) |
| Self-employment, current job |  | 0.15*** |  | 0.16*** |  | -0.018 |  | 0.066* |
|  |  | (0.000) |  | (0.000) |  | (0.543) |  | (0.042) |
| [1] Primary school |  | 0.10 |  | 0.37*** |  | 0.013 |  | 0.24*** |
|  |  | (0.081) |  | (0.000) |  | (0.793) |  | (0.000) |
| [2] Lower secondary school |  | 0.14* |  | 0.46*** |  | 0.011 |  | 0.32*** |
|  |  | (0.019) |  | (0.000) |  | (0.828) |  | (0.000) |
| [3] Upper secondary school |  | 0.18** |  | 0.58*** |  | 0.029 |  | 0.42*** |
|  |  | (0.002) |  | (0.000) |  | (0.568) |  | (0.000) |
| [4] Post-secondary non-tertiary education |  | 0.25*** |  | 0.69*** |  | 0.053 |  | 0.49*** |
|  |  | (0.000) |  | (0.000) |  | (0.359) |  | (0.000) |
| [5] First stage tertiary education |  | 0.26*** |  | 0.64*** |  | 0.0021 |  | 0.42*** |
|  |  | (0.000) |  | (0.000) |  | (0.967) |  | (0.000) |
| [6] Second stage tertiary education |  | 0.43*** |  | 0.77*** |  | 0.016 |  | 0.42*** |
|  |  | (0.000) |  | (0.000) |  | (0.856) |  | (0.000) |
| [1] Fair |  | 1.03*** |  | 1.12*** |  | 0.15*** |  | 1.24*** |
|  |  | (0.000) |  | (0.000) |  | (0.000) |  | (0.000) |
| [2] Good |  | 1.52*** |  | 1.80*** |  | 0.17*** |  | 1.96*** |
|  |  | (0.000) |  | (0.000) |  | (0.000) |  | (0.000) |
| [3] Very good |  | 1.86*** |  | 2.18*** |  | 0.31*** |  | 2.28*** |
|  |  | (0.000) |  | (0.000) |  | (0.000) |  | (0.000) |
| [4] Excellent |  | 2.18*** |  | 2.50*** |  | 0.45*** |  | 2.40*** |
|  |  | (0.000) |  | (0.000) |  | (0.000) |  | (0.000) |
| Drugs for depression |  | -0.49*** |  | -0.61*** |  | -0.092*** |  | -1.20*** |
|  |  | (0.000) |  | (0.000) |  | (0.000) |  | (0.000) |
| [1] Middle income |  | 0.16*** |  | 0.19*** |  | 0.018 |  | 0.082** |
|  |  | (0.000) |  | (0.000) |  | (0.429) |  | (0.002) |
| [2] Upper middle income |  | 0.23*** |  | 0.21*** |  | 0.028 |  | 0.067** |
|  |  | (0.000) |  | (0.000) |  | (0.206) |  | (0.008) |
| [3] High income |  | 0.24*** |  | 0.25*** |  | 0.018 |  | 0.059* |
|  |  | (0.000) |  | (0.000) |  | (0.402) |  | (0.013) |
| _cons | 6.95*** | 3.91*** | 4.41*** | 0.86* | 9.18*** | 8.73*** | 5.10*** | 2.64*** |
|  | (0.000) | (0.000) | (0.000) | (0.011) | (0.000) | (0.000) | (0.000) | (0.000) |
| N | 52248 | 46969 | 50512 | 45539 | 52513 | 47161 | 51941 | 46690 |
| R² | 0.12 | 0.24 | 0.19 | 0.37 | 0.03 | 0.04 | 0.10 | 0.31 |
| adjusted R² | 0.12 | 0.24 | 0.19 | 0.37 | 0.03 | 0.04 | 0.10 | 0.31 |
