# Supplementary material for: Marriage, parenthood and social network: Subjective well-being and mental health in old age
Source: PLoS One. 2019 Jul 24;14(7):e0218704. doi: 10.1371/journal.pone.0218704 (PMC6656342; doi:10.1371/journal.pone.0218704)
Supplement: S5 Table — (DOCX) [file pone.0218704.s010.docx]

**S5 Table. Regressing well-being and mental health on family status for all countries, male respondents**

|  | Life satisfaction | | CASP quality of life (0-10) | | Network satisfaction | | Lack of depressive symptoms (EURO-D) | |
| --- | --- | --- | --- | --- | --- | --- | --- | --- |
|  | A | B | A | B | A | B | A | B |
| [1] Having 1 child | 0.57*** | 0.49*** | 0.28*** | 0.18** | 0.40*** | 0.36*** | 0.30*** | 0.065 |
|  | (0.000) | (0.000) | (0.000) | (0.001) | (0.000) | (0.000) | (0.000) | (0.239) |
| [2] Having 2 children | 0.069 | -0.010 | 0.14** | 0.035 | 0.16*** | 0.12* | 0.035 | 0.016 |
|  | (0.167) | (0.840) | (0.004) | (0.455) | (0.000) | (0.013) | (0.469) | (0.729) |
| [3] Having 3 or more children | 0.24*** | 0.097* | 0.29*** | 0.13** | 0.16*** | 0.10* | 0.16*** | 0.094* |
|  | (0.000) | (0.040) | (0.000) | (0.004) | (0.000) | (0.021) | (0.000) | (0.032) |
| Number of children in same HH | 0.18*** | 0.029 | 0.24*** | 0.056 | 0.13** | 0.067 | 0.059 | -0.010 |
|  | (0.000) | (0.573) | (0.000) | (0.249) | (0.004) | (0.179) | (0.235) | (0.835) |
| Number of grandchildren | -0.0084 | -0.027 | -0.094*** | -0.11*** | -0.023 | -0.027 | -0.018 | -0.032 |
|  | (0.648) | (0.134) | (0.000) | (0.000) | (0.166) | (0.107) | (0.309) | (0.059) |
| Married/registered partnership | -0.0030 | 0.015** | -0.025*** | -0.0018 | 0.015*** | 0.018*** | -0.015** | -0.000094 |
|  | (0.566) | (0.004) | (0.000) | (0.707) | (0.001) | (0.000) | (0.004) | (0.985) |
| **Controls** |  |  |  |  |  |  |  |  |
| Age at interview | 0.055*** | 0.085*** | 0.14*** | 0.15*** | -0.031* | -0.029 | 0.13*** | 0.12*** |
|  | (0.001) | (0.000) | (0.000) | (0.000) | (0.024) | (0.066) | (0.000) | (0.000) |
| Age at interview, squared | -0.00038** | -0.00046*** | -0.0012*** | -0.0011*** | 0.00023* | 0.00024* | -0.0011*** | -0.00088*** |
|  | (0.002) | (0.000) | (0.000) | (0.000) | (0.023) | (0.036) | (0.000) | (0.000) |
| sh_country==[2]BEL | -0.46*** | -0.45*** | -0.73*** | -0.68*** | -0.59*** | -0.61*** | -0.34*** | -0.26*** |
|  | (0.000) | (0.000) | (0.000) | (0.000) | (0.000) | (0.000) | (0.000) | (0.000) |
| sh_country==[3]CHE | 0.12* | -0.14** | 0.23*** | -0.041 | -0.29*** | -0.39*** | 0.036 | -0.19*** |
|  | (0.013) | (0.007) | (0.000) | (0.399) | (0.000) | (0.000) | (0.476) | (0.000) |
| sh_country==[4]CZE | -0.93*** | -0.55*** | -1.42*** | -0.97*** | -0.34*** | -0.28*** | -0.12* | 0.23*** |
|  | (0.000) | (0.000) | (0.000) | (0.000) | (0.000) | (0.000) | (0.017) | (0.000) |
| sh_country==[5]DEU | -0.55*** | -0.46*** | -0.30*** | -0.20** | -0.41*** | -0.41*** | -0.13 | -0.040 |
|  | (0.000) | (0.000) | (0.000) | (0.003) | (0.000) | (0.000) | (0.053) | (0.547) |
| sh_country==[6]DNK | 0.21*** | -0.062 | 0.18** | -0.12* | 0.12* | 0.020 | 0.19*** | 0.0060 |
|  | (0.000) | (0.263) | (0.001) | (0.015) | (0.015) | (0.704) | (0.001) | (0.910) |
| sh_country==[7]ESP | -0.62*** | -0.32*** | -0.89*** | -0.42*** | -0.29*** | -0.21*** | -0.23*** | 0.053 |
|  | (0.000) | (0.000) | (0.000) | (0.000) | (0.000) | (0.000) | (0.000) | (0.378) |
| sh_country==[8]EST | -1.68*** | -1.35*** | -1.36*** | -0.91*** | -0.47*** | -0.43*** | -0.89*** | -0.44*** |
|  | (0.000) | (0.000) | (0.000) | (0.000) | (0.000) | (0.000) | (0.000) | (0.000) |
| sh_country==[9]FRA | -0.96*** | -0.82*** | -0.46*** | -0.28*** | -0.56*** | -0.56*** | -0.51*** | -0.32*** |
|  | (0.000) | (0.000) | (0.000) | (0.000) | (0.000) | (0.000) | (0.000) | (0.000) |
| sh_country==[10]HUN | -1.55*** | -1.02*** | -1.32*** | -0.65*** | -0.16** | -0.10 | -0.71*** | -0.21** |
|  | (0.000) | (0.000) | (0.000) | (0.000) | (0.004) | (0.115) | (0.000) | (0.001) |
| sh_country==[11]ITA | -0.64*** | -0.51*** | -1.52*** | -1.30*** | -0.37*** | -0.35*** | -0.40*** | -0.29*** |
|  | (0.000) | (0.000) | (0.000) | (0.000) | (0.000) | (0.000) | (0.000) | (0.000) |
| sh_country==[12]NLD | -0.24*** | -0.36*** | 0.26*** | 0.15** | -0.54*** | -0.59*** | 0.23*** | 0.13* |
|  | (0.000) | (0.000) | (0.000) | (0.003) | (0.000) | (0.000) | (0.000) | (0.011) |
| sh_country==[13]POL | -0.85*** | -0.34*** | -1.13*** | -0.48*** | -0.23** | -0.15 | -0.84*** | -0.33*** |
|  | (0.000) | (0.000) | (0.000) | (0.000) | (0.002) | (0.051) | (0.000) | (0.000) |
| sh_country==[14]PRT | -1.06*** | -0.58*** | -2.02*** | -1.29*** | -0.063 | 0.14* | -0.76*** | -0.23** |
|  | (0.000) | (0.000) | (0.000) | (0.000) | (0.286) | (0.039) | (0.000) | (0.002) |
| sh_country==[15]SVN | -0.96*** | -0.66*** | -0.13* | 0.25*** | -0.40*** | -0.37*** | -0.31*** | -0.072 |
|  | (0.000) | (0.000) | (0.035) | (0.000) | (0.000) | (0.000) | (0.000) | (0.224) |
| sh_country==[16]SWE | 0.026 | -0.18** | -0.29*** | -0.45*** | -0.050 | -0.12 | 0.030 | -0.064 |
|  | (0.670) | (0.003) | (0.000) | (0.000) | (0.392) | (0.055) | (0.623) | (0.273) |
| Divorced/living separated |  | 0.0044 |  | 0.023 |  | -0.061 |  | -0.12 |
|  |  | (0.951) |  | (0.715) |  | (0.380) |  | (0.063) |
| Widowed |  | 0.085 |  | 0.10 |  | 0.0013 |  | -0.23** |
|  |  | (0.281) |  | (0.143) |  | (0.987) |  | (0.001) |
| [1] Suburbs of big city |  | 0.0081 |  | 0.022 |  | 0.071 |  | -0.060 |
|  |  | (0.858) |  | (0.595) |  | (0.092) |  | (0.152) |
| [2] Large town |  | 0.042 |  | 0.057 |  | 0.12** |  | -0.065 |
|  |  | (0.318) |  | (0.132) |  | (0.002) |  | (0.094) |
| [3] Small town |  | 0.098* |  | 0.099** |  | 0.14*** |  | 0.049 |
|  |  | (0.013) |  | (0.005) |  | (0.000) |  | (0.165) |
| [4] Rural area/village |  | 0.060 |  | 0.085* |  | 0.13*** |  | 0.034 |
|  |  | (0.113) |  | (0.011) |  | (0.000) |  | (0.315) |
| Employment, current job |  | 0.29*** |  | 0.21*** |  | 0.047 |  | 0.13*** |
|  |  | (0.000) |  | (0.000) |  | (0.125) |  | (0.000) |
| Self-employment, current job |  | 0.21*** |  | 0.22*** |  | 0.023 |  | 0.074 |
|  |  | (0.000) |  | (0.000) |  | (0.574) |  | (0.059) |
| [1] Primary school |  | 0.23* |  | 0.40*** |  | 0.077 |  | 0.18* |
|  |  | (0.011) |  | (0.000) |  | (0.350) |  | (0.034) |
| [2] Lower secondary school |  | 0.24* |  | 0.50*** |  | 0.10 |  | 0.26** |
|  |  | (0.010) |  | (0.000) |  | (0.233) |  | (0.003) |
| [3] Upper secondary school |  | 0.27** |  | 0.62*** |  | 0.11 |  | 0.29** |
|  |  | (0.003) |  | (0.000) |  | (0.177) |  | (0.001) |
| [4] Post-secondary non-tertiary education |  | 0.37*** |  | 0.66*** |  | 0.17 |  | 0.28** |
|  |  | (0.000) |  | (0.000) |  | (0.080) |  | (0.005) |
| [5] First stage tertiary education |  | 0.32*** |  | 0.67*** |  | 0.11 |  | 0.26** |
|  |  | (0.000) |  | (0.000) |  | (0.210) |  | (0.003) |
| [6] Second stage tertiary education |  | 0.45*** |  | 0.77*** |  | 0.013 |  | 0.22 |
|  |  | (0.000) |  | (0.000) |  | (0.922) |  | (0.079) |
| [1] Fair |  | 1.05*** |  | 1.12*** |  | 0.13** |  | 1.20*** |
|  |  | (0.000) |  | (0.000) |  | (0.002) |  | (0.000) |
| [2] Good |  | 1.49*** |  | 1.76*** |  | 0.15*** |  | 1.84*** |
|  |  | (0.000) |  | (0.000) |  | (0.000) |  | (0.000) |
| [3] Very good |  | 1.83*** |  | 2.14*** |  | 0.30*** |  | 2.12*** |
|  |  | (0.000) |  | (0.000) |  | (0.000) |  | (0.000) |
| [4] Excellent |  | 2.11*** |  | 2.45*** |  | 0.43*** |  | 2.22*** |
|  |  | (0.000) |  | (0.000) |  | (0.000) |  | (0.000) |
| Drugs for depression |  | -0.44*** |  | -0.57*** |  | -0.081* |  | -1.22*** |
|  |  | (0.000) |  | (0.000) |  | (0.039) |  | (0.000) |
| [1] Middle income |  | 0.23*** |  | 0.26*** |  | 0.061 |  | 0.13*** |
|  |  | (0.000) |  | (0.000) |  | (0.096) |  | (0.000) |
| [2] Upper middle income |  | 0.29*** |  | 0.31*** |  | 0.041 |  | 0.16*** |
|  |  | (0.000) |  | (0.000) |  | (0.253) |  | (0.000) |
| [3] High income |  | 0.26*** |  | 0.31*** |  | 0.076* |  | 0.11*** |
|  |  | (0.000) |  | (0.000) |  | (0.023) |  | (0.001) |
| _cons | 5.79*** | 2.29*** | 3.65*** | 0.081 | 9.66*** | 9.11*** | 4.40*** | 2.41*** |
|  | (0.000) | (0.000) | (0.000) | (0.876) | (0.000) | (0.000) | (0.000) | (0.000) |
| N | 22847 | 20648 | 22149 | 20067 | 22962 | 20735 | 22705 | 20518 |
| R² | 0.12 | 0.25 | 0.18 | 0.36 | 0.04 | 0.05 | 0.06 | 0.27 |
| adjusted R² | 0.12 | 0.25 | 0.18 | 0.36 | 0.04 | 0.04 | 0.06 | 0.27 |
