# Supplementary material for: Marriage, parenthood and social network: Subjective well-being and mental health in old age
Source: PLoS One. 2019 Jul 24;14(7):e0218704. doi: 10.1371/journal.pone.0218704 (PMC6656342; doi:10.1371/journal.pone.0218704)
Supplement: S6 Table — (DOCX) [file pone.0218704.s011.docx]

**S6Table. Regressing well-being and mental health on family status for all countries, female respondents**

|  | Life satisfaction | | CASP quality of life (0-10) | | Network satisfaction | | Lack of depressive symptoms (EURO-D) | |
| --- | --- | --- | --- | --- | --- | --- | --- | --- |
|  | A | B | A | B | A | B | A | B |
| [1] Having 1 child | 0.55*** | 0.39*** | 0.30*** | 0.21*** | 0.11*** | 0.18*** | 0.27*** | 0.034 |
|  | (0.000) | (0.000) | (0.000) | (0.000) | (0.000) | (0.000) | (0.000) | (0.531) |
| [2] Having 2 children | -0.052 | -0.030 | 0.0058 | 0.027 | 0.24*** | 0.20*** | -0.11* | -0.030 |
|  | (0.243) | (0.521) | (0.892) | (0.512) | (0.000) | (0.000) | (0.020) | (0.518) |
| [3] Having 3 or more children | 0.12** | 0.13** | 0.11** | 0.091* | 0.29*** | 0.25*** | 0.059 | 0.11* |
|  | (0.004) | (0.005) | (0.008) | (0.023) | (0.000) | (0.000) | (0.195) | (0.018) |
| Number of children in same HH | 0.062 | 0.082 | 0.044 | 0.049 | 0.29*** | 0.25*** | -0.013 | 0.058 |
|  | (0.201) | (0.102) | (0.334) | (0.276) | (0.000) | (0.000) | (0.807) | (0.253) |
| Number of grandchildren | -0.056** | -0.081*** | -0.10*** | -0.13*** | -0.021 | -0.027 | -0.021 | -0.028 |
|  | (0.004) | (0.000) | (0.000) | (0.000) | (0.150) | (0.083) | (0.310) | (0.151) |
| Married/registered partnership | -0.0051 | 0.0077 | -0.019*** | 0.0012 | 0.013*** | 0.016*** | -0.023*** | -0.0040 |
|  | (0.311) | (0.117) | (0.000) | (0.774) | (0.000) | (0.000) | (0.000) | (0.400) |
| **Controls** |  |  |  |  |  |  |  |  |
| Age at interview | -0.00017 | 0.013 | 0.10*** | 0.12*** | -0.0033 | -0.0031 | 0.11*** | 0.099*** |
|  | (0.991) | (0.407) | (0.000) | (0.000) | (0.760) | (0.804) | (0.000) | (0.000) |
| Age at interview, squared | 0.000024 | 0.000065 | -0.00095*** | -0.00089*** | 0.0000030 | 0.0000075 | -0.00091*** | -0.00067*** |
|  | (0.819) | (0.553) | (0.000) | (0.000) | (0.971) | (0.934) | (0.000) | (0.000) |
| sh_country==[2]BEL | -0.60*** | -0.44*** | -0.82*** | -0.63*** | -0.70*** | -0.66*** | -0.60*** | -0.31*** |
|  | (0.000) | (0.000) | (0.000) | (0.000) | (0.000) | (0.000) | (0.000) | (0.000) |
| sh_country==[3]CHE | 0.072 | -0.11* | 0.29*** | 0.092* | -0.41*** | -0.39*** | -0.034 | -0.20*** |
|  | (0.110) | (0.019) | (0.000) | (0.037) | (0.000) | (0.000) | (0.496) | (0.000) |
| sh_country==[4]CZE | -1.04*** | -0.68*** | -1.45*** | -1.02*** | -0.47*** | -0.43*** | -0.32*** | 0.061 |
|  | (0.000) | (0.000) | (0.000) | (0.000) | (0.000) | (0.000) | (0.000) | (0.215) |
| sh_country==[5]DEU | -0.55*** | -0.46*** | -0.24*** | -0.11 | -0.44*** | -0.42*** | -0.35*** | -0.22*** |
|  | (0.000) | (0.000) | (0.000) | (0.060) | (0.000) | (0.000) | (0.000) | (0.001) |
| sh_country==[6]DNK | 0.32*** | 0.093 | 0.30*** | 0.039 | 0.027 | -0.0022 | 0.14* | -0.054 |
|  | (0.000) | (0.082) | (0.000) | (0.414) | (0.501) | (0.959) | (0.017) | (0.350) |
| sh_country==[7]ESP | -0.87*** | -0.42*** | -1.27*** | -0.65*** | -0.31*** | -0.26*** | -1.06*** | -0.42*** |
|  | (0.000) | (0.000) | (0.000) | (0.000) | (0.000) | (0.000) | (0.000) | (0.000) |
| sh_country==[8]EST | -1.52*** | -1.15*** | -1.14*** | -0.71*** | -0.43*** | -0.34*** | -1.00*** | -0.45*** |
|  | (0.000) | (0.000) | (0.000) | (0.000) | (0.000) | (0.000) | (0.000) | (0.000) |
| sh_country==[9]FRA | -1.06*** | -0.86*** | -0.60*** | -0.30*** | -0.60*** | -0.55*** | -0.81*** | -0.46*** |
|  | (0.000) | (0.000) | (0.000) | (0.000) | (0.000) | (0.000) | (0.000) | (0.000) |
| sh_country==[10]HUN | -1.64*** | -1.03*** | -1.43*** | -0.69*** | -0.17*** | -0.086 | -1.13*** | -0.43*** |
|  | (0.000) | (0.000) | (0.000) | (0.000) | (0.000) | (0.092) | (0.000) | (0.000) |
| sh_country==[11]ITA | -0.82*** | -0.52*** | -1.81*** | -1.45*** | -0.40*** | -0.36*** | -0.79*** | -0.43*** |
|  | (0.000) | (0.000) | (0.000) | (0.000) | (0.000) | (0.000) | (0.000) | (0.000) |
| sh_country==[12]NLD | -0.33*** | -0.34*** | 0.22*** | 0.23*** | -0.68*** | -0.69*** | -0.039 | 0.031 |
|  | (0.000) | (0.000) | (0.000) | (0.000) | (0.000) | (0.000) | (0.490) | (0.569) |
| sh_country==[13]POL | -0.99*** | -0.38*** | -1.25*** | -0.57*** | -0.25*** | -0.17** | -1.17*** | -0.54*** |
|  | (0.000) | (0.000) | (0.000) | (0.000) | (0.000) | (0.008) | (0.000) | (0.000) |
| sh_country==[14]PRT | -1.51*** | -0.70*** | -2.28*** | -1.34*** | -0.12* | 0.076 | -1.50*** | -0.45*** |
|  | (0.000) | (0.000) | (0.000) | (0.000) | (0.016) | (0.161) | (0.000) | (0.000) |
| sh_country==[15]SVN | -0.85*** | -0.54*** | -0.20*** | 0.20*** | -0.47*** | -0.40*** | -0.44*** | -0.15* |
|  | (0.000) | (0.000) | (0.000) | (0.000) | (0.000) | (0.000) | (0.000) | (0.013) |
| sh_country==[16]SWE | 0.040 | -0.035 | -0.13* | -0.20*** | -0.10* | -0.13** | 0.036 | 0.026 |
|  | (0.495) | (0.542) | (0.017) | (0.000) | (0.025) | (0.007) | (0.556) | (0.655) |
| Divorced/living separated |  | -0.18** |  | -0.10 |  | 0.0076 |  | -0.13* |
|  |  | (0.004) |  | (0.057) |  | (0.890) |  | (0.033) |
| Widowed |  | 0.015 |  | 0.099 |  | 0.14* |  | -0.13* |
|  |  | (0.796) |  | (0.058) |  | (0.010) |  | (0.029) |
| [1] Suburbs of big city |  | 0.0035 |  | 0.011 |  | 0.0056 |  | -0.098* |
|  |  | (0.933) |  | (0.774) |  | (0.868) |  | (0.022) |
| [2] Large town |  | 0.032 |  | -0.012 |  | 0.039 |  | -0.070 |
|  |  | (0.399) |  | (0.727) |  | (0.214) |  | (0.069) |
| [3] Small town |  | 0.11** |  | 0.036 |  | 0.053 |  | 0.00086 |
|  |  | (0.003) |  | (0.256) |  | (0.064) |  | (0.981) |
| [4] Rural area/village |  | 0.056 |  | 0.0087 |  | -0.034 |  | -0.021 |
|  |  | (0.108) |  | (0.777) |  | (0.225) |  | (0.544) |
| Employment, current job |  | 0.078* |  | 0.13*** |  | 0.00029 |  | 0.055 |
|  |  | (0.013) |  | (0.000) |  | (0.991) |  | (0.083) |
| Self-employment, current job |  | 0.084 |  | 0.087 |  | -0.086 |  | 0.070 |
|  |  | (0.113) |  | (0.074) |  | (0.065) |  | (0.215) |
| [1] Primary school |  | 0.019 |  | 0.34*** |  | -0.018 |  | 0.25*** |
|  |  | (0.802) |  | (0.000) |  | (0.773) |  | (0.001) |
| [2] Lower secondary school |  | 0.060 |  | 0.42*** |  | -0.041 |  | 0.33*** |
|  |  | (0.433) |  | (0.000) |  | (0.518) |  | (0.000) |
| [3] Upper secondary school |  | 0.11 |  | 0.53*** |  | -0.020 |  | 0.48*** |
|  |  | (0.158) |  | (0.000) |  | (0.752) |  | (0.000) |
| [4] Post-secondary non-tertiary education |  | 0.15 |  | 0.69*** |  | -0.016 |  | 0.62*** |
|  |  | (0.083) |  | (0.000) |  | (0.829) |  | (0.000) |
| [5] First stage tertiary education |  | 0.23** |  | 0.60*** |  | -0.073 |  | 0.51*** |
|  |  | (0.004) |  | (0.000) |  | (0.267) |  | (0.000) |
| [6] Second stage tertiary education |  | 0.45** |  | 0.76*** |  | 0.097 |  | 0.61*** |
|  |  | (0.001) |  | (0.000) |  | (0.384) |  | (0.000) |
| [1] Fair |  | 1.01*** |  | 1.12*** |  | 0.17*** |  | 1.26*** |
|  |  | (0.000) |  | (0.000) |  | (0.000) |  | (0.000) |
| [2] Good |  | 1.54*** |  | 1.82*** |  | 0.19*** |  | 2.03*** |
|  |  | (0.000) |  | (0.000) |  | (0.000) |  | (0.000) |
| [3] Very good |  | 1.88*** |  | 2.21*** |  | 0.33*** |  | 2.41*** |
|  |  | (0.000) |  | (0.000) |  | (0.000) |  | (0.000) |
| [4] Excellent |  | 2.23*** |  | 2.53*** |  | 0.47*** |  | 2.55*** |
|  |  | (0.000) |  | (0.000) |  | (0.000) |  | (0.000) |
| Drugs for depression |  | -0.51*** |  | -0.62*** |  | -0.10*** |  | -1.17*** |
|  |  | (0.000) |  | (0.000) |  | (0.000) |  | (0.000) |
| Middle income |  | 0.10** |  | 0.14*** |  | -0.0070 |  | 0.054 |
|  |  | (0.004) |  | (0.000) |  | (0.816) |  | (0.145) |
| Upper middle income |  | 0.19*** |  | 0.13*** |  | 0.024 |  | 0.0055 |
|  |  | (0.000) |  | (0.000) |  | (0.409) |  | (0.877) |
| High income |  | 0.23*** |  | 0.21*** |  | -0.015 |  | 0.037 |
|  |  | (0.000) |  | (0.000) |  | (0.583) |  | (0.271) |
| _cons | 7.81*** | 5.16*** | 4.88*** | 1.40** | 9.12*** | 8.85*** | 5.02*** | 2.37*** |
|  | (0.000) | (0.000) | (0.000) | (0.002) | (0.000) | (0.000) | (0.000) | (0.000) |
| N | 29401 | 26321 | 28363 | 25472 | 29551 | 26426 | 29236 | 26172 |
| R² | 0.12 | 0.24 | 0.20 | 0.38 | 0.03 | 0.04 | 0.08 | 0.29 |
| adjusted R² | 0.12 | 0.24 | 0.20 | 0.38 | 0.03 | 0.04 | 0.07 | 0.29 |
