# Supplementary material for: Marriage, parenthood and social network: Subjective well-being and mental health in old age
Source: PLoS One. 2019 Jul 24;14(7):e0218704. doi: 10.1371/journal.pone.0218704 (PMC6656342; doi:10.1371/journal.pone.0218704)
Supplement: S7 Table — (DOCX) [file pone.0218704.s012.docx]

**S7 Table. Regressing well-being and mental health on family status for each country**

| Country | Married | | | | 1 child | | | | 2 children | | | | 3 or more children | | | | Resident children | | | | Grandchildren | | | |
| --- | --- | --- | --- | --- | --- | --- | --- | --- | --- | --- | --- | --- | --- | --- | --- | --- | --- | --- | --- | --- | --- | --- | --- | --- |
|  | (I) | (II) | (III) | (IV) | (I) | (II) | (III) | (IV) | (I) | (II) | (III) | (IV) | (I) | (II) | (III) | (IV) | (I) | (II) | (III) | (IV) | (I) | (II) | (III) | (IV) |
| Austria | + | + | + | + | (+) | (+) | + | o | + | (+) | + | (+) | o | (+) | + | o | o | - | o | o | + | (-) | (+) | o |
| Belgium | + | + | + | + | o | o | (+) | o | o | o | + | o | o | o | + | o | o | o | o | o | o | o | o | o |
| Czech Republic | + | + | + | + | o | o | o | o | (+) | o | o | o | o | o | o | o | o | o | o | o | o | o | + | o |
| Denmark | + | + | + | o | o | o | o | o | + | o | o | o | (+) | o | o | o | o | o | o | o | o | o | o | o |
| Estonia | + | + | + | + | o | (+) | + | o | + | + | + | + | + | + | + | + | - | - | o | - | o | o | + | - |
| France | + | + | o | + | - | - | + | - | o | o | + | o | o | o | + | o | o | - | o | o | (-) | - | o | o |
| Germany | + | + | + | o | o | o | o | o | o | o | o | o | o | o | o | o | - | o | o | o | - | o | o | - |
| Hungary | + | + | + | + | o | o | + | - | o | + | + | o | o | o | + | (-) | o | - | o | o | o | - | o | - |
| Italy | + | + | + | + | o | o | + | - | o | o | o | - | o | (-) | o | - | o | - | o | o | o | - | o | - |
| Netherlands | + | + | o | + | o | o | (+) | o | + | o | (+) | o | + | o | o | o | o | o | o | o | o | o | (+) | o |
| Poland | + | (+) | + | o | o | o | o | o | o | o | o | o | o | o | o | o | o | - | o | o | o | o | + | o |
| Portugal | + | o | + | o | o | o | o | o | (+) | (+) | o | o | (+) | + | o | o | o | - | o | o | - | - | o | - |
| Slovenia | + | + | (+) | + | o | o | + | o | o | o | o | o | o | o | o | o | o | o | o | o | o | (-) | + | (-) |
| Spain | + | + | + | + | o | o | + | o | o | + | + | o | o | o | + | o | o | - | o | - | o | - | o | - |
| Sweden | + | + | + | o | o | + | + | o | o | + | (+) | + | o | + | + | o | o | o | (+) | o | o | o | o | o |
| Switzerland | + | + | o | + | o | o | o | o | o | o | + | (+) | + | + | + | o | o | o | o | o | o | o | o | o |

+/ - indicates positive/negative significant effect at 5% significance level; additionally (+),(-) indicates positive, or negative effect at 10% significance level, and o indicates that there is no significant effect at 10% level. Dependent Variables: (I) Life satisfaction, (II) CASP-12, (III) Network satisfaction, (IV) EURO-D. (I)-(IV) country-specific OLS Regression. Controls A: female, age, age^2^, country dummy. Children: A dummy variable for having no children (excluded category), one child, two children, and three or more children. Resident children: Number of children living with their parents. If a respondent has no children then the value is set to 0. Grandchildren: Number of grandchildren, Married: Dummy variable if respondent is married or in registered partnership. Excluded category: Married but living separated from a spouse, never married, divorced, widowed.
