# Supplementary material for: Marriage, parenthood and social network: Subjective well-being and mental health in old age
Source: PLoS One. 2019 Jul 24;14(7):e0218704. doi: 10.1371/journal.pone.0218704 (PMC6656342; doi:10.1371/journal.pone.0218704)
Supplement: S8 Table — (DOCX) [file pone.0218704.s013.docx]

**S8 Table. Regressing well-being and mental health on network types controlling for network size and family status for all countries, all respondents**

|  | Life satisfaction | | Quality of life (CASP-12) | | Network satisfaction | | Lack of depressive symptoms (EURO-D) | |
| --- | --- | --- | --- | --- | --- | --- | --- | --- |
|  | A | B | A | B | A | B | A | B |
| [1] Partner | 0.44*** | 0.46*** | 0.20*** | 0.20*** | 2.37*** | 2.47*** | 0.30*** | 0.32*** |
|  | (0.000) | (0.000) | (0.000) | (0.000) | (0.000) | (0.000) | (0.000) | (0.000) |
| [2] Children | 0.21*** | 0.28*** | -0.082 | 0.028 | 2.42*** | 2.50*** | 0.021 | 0.19*** |
|  | (0.001) | (0.000) | (0.123) | (0.572) | (0.000) | (0.000) | (0.723) | (0.001) |
| [3] Other Relatives | 0.21*** | 0.26*** | 0.066 | 0.12* | 2.14*** | 2.24*** | 0.0040 | 0.098 |
|  | (0.000) | (0.000) | (0.219) | (0.014) | (0.000) | (0.000) | (0.946) | (0.086) |
| [4] Family | 0.30*** | 0.37*** | 0.037 | 0.12* | 2.30*** | 2.40*** | 0.12* | 0.25*** |
|  | (0.000) | (0.000) | (0.477) | (0.014) | (0.000) | (0.000) | (0.034) | (0.000) |
| [5] Friends | 0.21*** | 0.24*** | 0.10 | 0.15** | 2.03*** | 2.13*** | 0.0013 | 0.095 |
|  | (0.000) | (0.000) | (0.051) | (0.002) | (0.000) | (0.000) | (0.982) | (0.088) |
| [6] Diverse | 0.12 | 0.23*** | -0.083 | 0.045 | 1.97*** | 2.07*** | -0.12* | 0.045 |
|  | (0.055) | (0.000) | (0.135) | (0.385) | (0.000) | (0.000) | (0.044) | (0.440) |
| Size of social network | 0.097*** | 0.065*** | 0.11*** | 0.069*** | 0.057*** | 0.054*** | 0.053*** | 0.017** |
|  | (0.000) | (0.000) | (0.000) | (0.000) | (0.000) | (0.000) | (0.000) | (0.004) |
| Married/registered partnership | 0.48*** | 0.38*** | 0.24*** | 0.17*** | 0.10*** | 0.13*** | 0.20*** | -0.022 |
|  | (0.000) | (0.000) | (0.000) | (0.000) | (0.000) | (0.000) | (0.000) | (0.576) |
| [1] Having 1 child | -0.018 | -0.054 | 0.087** | 0.035 | 0.10*** | 0.074* | -0.056 | -0.047 |
|  | (0.589) | (0.125) | (0.006) | (0.260) | (0.000) | (0.014) | (0.105) | (0.172) |
| [2] Having 2 children | 0.13*** | 0.062 | 0.19*** | 0.097** | 0.095*** | 0.069* | 0.075* | 0.055 |
|  | (0.000) | (0.064) | (0.000) | (0.001) | (0.000) | (0.019) | (0.023) | (0.092) |
| [3] Having 3 or more children | 0.055 | 0.0015 | 0.12*** | 0.036 | 0.077** | 0.046 | -0.018 | -0.026 |
|  | (0.124) | (0.967) | (0.000) | (0.280) | (0.008) | (0.149) | (0.619) | (0.473) |
| Number of resident children | -0.027* | -0.050*** | -0.094*** | -0.12*** | -0.015 | -0.021 | -0.015 | -0.024 |
|  | (0.045) | (0.000) | (0.000) | (0.000) | (0.135) | (0.057) | (0.281) | (0.058) |
| Number of grandchildren | -0.0054 | 0.0093** | -0.021*** | -0.00045 | 0.012*** | 0.015*** | -0.021*** | -0.0032 |
|  | (0.138) | (0.009) | (0.000) | (0.884) | (0.000) | (0.000) | (0.000) | (0.349) |
| **Controls** |  |  |  |  |  |  |  |  |
| Female | -0.044** | 0.041** | -0.17*** | -0.060*** | 0.11*** | 0.11*** | -0.64*** | -0.50*** |
|  | (0.005) | (0.009) | (0.000) | (0.000) | (0.000) | (0.000) | (0.000) | (0.000) |
| Age at interview | 0.020 | 0.041*** | 0.11*** | 0.13*** | -0.013 | -0.011 | 0.12*** | 0.11*** |
|  | (0.064) | (0.000) | (0.000) | (0.000) | (0.099) | (0.219) | (0.000) | (0.000) |
| Age at interview, squared | -0.00011 | -0.00013 | -0.00099*** | -0.00095*** | 0.000088 | 0.000086 | -0.00097*** | -0.00076*** |
|  | (0.161) | (0.110) | (0.000) | (0.000) | (0.140) | (0.192) | (0.000) | (0.000) |
| sh_country==[2]BEL | -0.53*** | -0.44*** | -0.78*** | -0.65*** | -0.63*** | -0.63*** | -0.49*** | -0.29*** |
|  | (0.000) | (0.000) | (0.000) | (0.000) | (0.000) | (0.000) | (0.000) | (0.000) |
| sh_country==[3]CHE | 0.089** | -0.12*** | 0.26*** | 0.026 | -0.37*** | -0.41*** | -0.022 | -0.22*** |
|  | (0.008) | (0.000) | (0.000) | (0.424) | (0.000) | (0.000) | (0.533) | (0.000) |
| sh_country==[4]CZE | -0.94*** | -0.60*** | -1.38*** | -0.97*** | -0.37*** | -0.35*** | -0.24*** | 0.12** |
|  | (0.000) | (0.000) | (0.000) | (0.000) | (0.000) | (0.000) | (0.000) | (0.001) |
| sh_country==[5]DEU | -0.56*** | -0.47*** | -0.26*** | -0.15** | -0.45*** | -0.43*** | -0.28*** | -0.16*** |
|  | (0.000) | (0.000) | (0.000) | (0.001) | (0.000) | (0.000) | (0.000) | (0.000) |
| sh_country==[6]DNK | 0.27*** | 0.033 | 0.26*** | -0.015 | 0.035 | -0.014 | 0.15*** | -0.038 |
|  | (0.000) | (0.398) | (0.000) | (0.660) | (0.272) | (0.685) | (0.000) | (0.335) |
| sh_country==[7]ESP | -0.75*** | -0.38*** | -1.07*** | -0.54*** | -0.33*** | -0.30*** | -0.70*** | -0.23*** |
|  | (0.000) | (0.000) | (0.000) | (0.000) | (0.000) | (0.000) | (0.000) | (0.000) |
| sh_country==[8]EST | -1.55*** | -1.21*** | -1.19*** | -0.77*** | -0.42*** | -0.36*** | -0.94*** | -0.44*** |
|  | (0.000) | (0.000) | (0.000) | (0.000) | (0.000) | (0.000) | (0.000) | (0.000) |
| sh_country==[9]FRA | -0.98*** | -0.82*** | -0.52*** | -0.29*** | -0.51*** | -0.50*** | -0.67*** | -0.38*** |
|  | (0.000) | (0.000) | (0.000) | (0.000) | (0.000) | (0.000) | (0.000) | (0.000) |
| sh_country==[10]HUN | -1.58*** | -1.03*** | -1.34*** | -0.67*** | -0.18*** | -0.12** | -0.94*** | -0.35*** |
|  | (0.000) | (0.000) | (0.000) | (0.000) | (0.000) | (0.001) | (0.000) | (0.000) |
| sh_country==[11]ITA | -0.67*** | -0.47*** | -1.60*** | -1.34*** | -0.25*** | -0.22*** | -0.58*** | -0.35*** |
|  | (0.000) | (0.000) | (0.000) | (0.000) | (0.000) | (0.000) | (0.000) | (0.000) |
| sh_country==[12]NLD | -0.28*** | -0.35*** | 0.25*** | 0.20*** | -0.64*** | -0.67*** | 0.075 | 0.063 |
|  | (0.000) | (0.000) | (0.000) | (0.000) | (0.000) | (0.000) | (0.058) | (0.095) |
| sh_country==[13]POL | -0.89*** | -0.35*** | -1.13*** | -0.50*** | -0.25*** | -0.18*** | -1.04*** | -0.49*** |
|  | (0.000) | (0.000) | (0.000) | (0.000) | (0.000) | (0.000) | (0.000) | (0.000) |
| sh_country==[14]PRT | -1.30*** | -0.66*** | -2.13*** | -1.32*** | -0.13*** | 0.036 | -1.19*** | -0.38*** |
|  | (0.000) | (0.000) | (0.000) | (0.000) | (0.000) | (0.364) | (0.000) | (0.000) |
| sh_country==[15]SVN | -0.82*** | -0.55*** | -0.091* | 0.27*** | -0.31*** | -0.26*** | -0.39*** | -0.13** |
|  | (0.000) | (0.000) | (0.024) | (0.000) | (0.000) | (0.000) | (0.000) | (0.002) |
| sh_country==[16]SWE | 0.045 | -0.094* | -0.17*** | -0.29*** | -0.14*** | -0.18*** | 0.019 | -0.034 |
|  | (0.288) | (0.027) | (0.000) | (0.000) | (0.000) | (0.000) | (0.667) | (0.416) |
| Divorced/living separated |  | -0.11* |  | -0.047 |  | -0.021 |  | -0.14** |
|  |  | (0.025) |  | (0.250) |  | (0.600) |  | (0.002) |
| Widowed |  | 0.072 |  | 0.13** |  | 0.091* |  | -0.15*** |
|  |  | (0.123) |  | (0.002) |  | (0.024) |  | (0.001) |
| [1] Suburbs of big city |  | 0.013 |  | 0.032 |  | 0.015 |  | -0.087** |
|  |  | (0.681) |  | (0.248) |  | (0.546) |  | (0.004) |
| [2] Large town |  | 0.047 |  | 0.037 |  | 0.077** |  | -0.074** |
|  |  | (0.098) |  | (0.139) |  | (0.001) |  | (0.008) |
| [3] Small town |  | 0.11*** |  | 0.080*** |  | 0.089*** |  | 0.018 |
|  |  | (0.000) |  | (0.001) |  | (0.000) |  | (0.490) |
| [4] Rural area/village |  | 0.069** |  | 0.066** |  | 0.033 |  | -0.0040 |
|  |  | (0.007) |  | (0.003) |  | (0.116) |  | (0.871) |
| Employment, current job |  | 0.17*** |  | 0.17*** |  | 0.025 |  | 0.097*** |
|  |  | (0.000) |  | (0.000) |  | (0.180) |  | (0.000) |
| Self-employment, current job |  | 0.15*** |  | 0.16*** |  | -0.021 |  | 0.072* |
|  |  | (0.000) |  | (0.000) |  | (0.475) |  | (0.026) |
| [1] Primary school |  | 0.087 |  | 0.35*** |  | -0.034 |  | 0.23*** |
|  |  | (0.135) |  | (0.000) |  | (0.454) |  | (0.000) |
| [2] Lower secondary school |  | 0.12* |  | 0.44*** |  | -0.044 |  | 0.32*** |
|  |  | (0.048) |  | (0.000) |  | (0.340) |  | (0.000) |
| [3] Upper secondary school |  | 0.15* |  | 0.54*** |  | -0.041 |  | 0.41*** |
|  |  | (0.012) |  | (0.000) |  | (0.380) |  | (0.000) |
| [4] Post-secondary non-tertiary education |  | 0.21** |  | 0.64*** |  | -0.018 |  | 0.49*** |
|  |  | (0.002) |  | (0.000) |  | (0.735) |  | (0.000) |
| [5] First stage tertiary education |  | 0.22*** |  | 0.58*** |  | -0.070 |  | 0.42*** |
|  |  | (0.000) |  | (0.000) |  | (0.139) |  | (0.000) |
| [6] Second stage tertiary education |  | 0.39*** |  | 0.71*** |  | -0.050 |  | 0.42*** |
|  |  | (0.000) |  | (0.000) |  | (0.545) |  | (0.000) |
| [1] Fair |  | 1.02*** |  | 1.11*** |  | 0.14*** |  | 1.24*** |
|  |  | (0.000) |  | (0.000) |  | (0.000) |  | (0.000) |
| [2] Good |  | 1.51*** |  | 1.78*** |  | 0.16*** |  | 1.95*** |
|  |  | (0.000) |  | (0.000) |  | (0.000) |  | (0.000) |
| [3] Very good |  | 1.84*** |  | 2.16*** |  | 0.30*** |  | 2.28*** |
|  |  | (0.000) |  | (0.000) |  | (0.000) |  | (0.000) |
| [4] Excellent |  | 2.17*** |  | 2.48*** |  | 0.44*** |  | 2.40*** |
|  |  | (0.000) |  | (0.000) |  | (0.000) |  | (0.000) |
| Drugs for depression |  | -0.50*** |  | -0.61*** |  | -0.094*** |  | -1.19*** |
|  |  | (0.000) |  | (0.000) |  | (0.000) |  | (0.000) |
| [1] Middle income |  | 0.15*** |  | 0.19*** |  | -0.0028 |  | 0.080** |
|  |  | (0.000) |  | (0.000) |  | (0.898) |  | (0.002) |
| [2] Upper middle income |  | 0.23*** |  | 0.21*** |  | 0.016 |  | 0.070** |
|  |  | (0.000) |  | (0.000) |  | (0.469) |  | (0.006) |
| [3] High income |  | 0.24*** |  | 0.24*** |  | 0.00069 |  | 0.061* |
|  |  | (0.000) |  | (0.000) |  | (0.973) |  | (0.010) |
| _cons | 6.61*** | 3.62*** | 4.20*** | 0.72* | 7.13*** | 6.70*** | 5.04*** | 2.55*** |
|  | (0.000) | (0.000) | (0.000) | (0.035) | (0.000) | (0.000) | (0.000) | (0.000) |
| N | 52248 | 46969 | 50512 | 45539 | 52513 | 47161 | 51941 | 46690 |
| R² | 0.13 | 0.25 | 0.20 | 0.38 | 0.12 | 0.13 | 0.10 | 0.31 |
| adjusted R² | 0.13 | 0.25 | 0.20 | 0.38 | 0.12 | 0.13 | 0.10 | 0.31 |
