# Supplementary material for: Marriage, parenthood and social network: Subjective well-being and mental health in old age
Source: PLoS One. 2019 Jul 24;14(7):e0218704. doi: 10.1371/journal.pone.0218704 (PMC6656342; doi:10.1371/journal.pone.0218704)
Supplement: S9 Table — (DOCX) [file pone.0218704.s014.docx]

**S9 Table. Regressing well-being and mental health on network types controlling for network size and family status for all countries, male respondents**

|  | Life satisfaction | | Quality of life (CASP-12) | | Network satisfaction | | Lack of depressive symptoms (EURO-D) | |
| --- | --- | --- | --- | --- | --- | --- | --- | --- |
|  | A | B | A | B | A | B | A | B |
| [1] Partner | 0.46*** | 0.44*** | 0.16* | 0.16* | 2.28*** | 2.39*** | 0.19** | 0.22** |
|  | (0.000) | (0.000) | (0.019) | (0.015) | (0.000) | (0.000) | (0.008) | (0.001) |
| [2] Children | 0.32*** | 0.33*** | 0.038 | 0.11 | 2.27*** | 2.38*** | 0.059 | 0.20* |
|  | (0.000) | (0.000) | (0.616) | (0.141) | (0.000) | (0.000) | (0.472) | (0.011) |
| [3] Other Relatives | 0.26** | 0.27*** | 0.098 | 0.14 | 1.94*** | 2.05*** | 0.0095 | 0.094 |
|  | (0.001) | (0.001) | (0.191) | (0.052) | (0.000) | (0.000) | (0.905) | (0.226) |
| [4] Family | 0.37*** | 0.40*** | 0.054 | 0.12 | 2.15*** | 2.26*** | 0.089 | 0.20** |
|  | (0.000) | (0.000) | (0.455) | (0.086) | (0.000) | (0.000) | (0.245) | (0.006) |
| [5] Friends | 0.31*** | 0.30*** | 0.15* | 0.17* | 1.80*** | 1.92*** | 0.014 | 0.084 |
|  | (0.000) | (0.000) | (0.037) | (0.021) | (0.000) | (0.000) | (0.856) | (0.271) |
| [6] Diverse | 0.23** | 0.23** | -0.0027 | 0.050 | 1.70*** | 1.81*** | -0.043 | 0.057 |
|  | (0.007) | (0.006) | (0.972) | (0.511) | (0.000) | (0.000) | (0.599) | (0.474) |
| Size of social network | 0.076*** | 0.052*** | 0.089*** | 0.055*** | 0.050*** | 0.048*** | 0.041*** | 0.016 |
|  | (0.000) | (0.000) | (0.000) | (0.000) | (0.000) | (0.000) | (0.000) | (0.081) |
| Married/registered partnership | 0.50*** | 0.43*** | 0.25*** | 0.17** | 0.22*** | 0.15** | 0.24*** | 0.019 |
|  | (0.000) | (0.000) | (0.000) | (0.002) | (0.000) | (0.007) | (0.000) | (0.739) |
| [1] Having 1 child | 0.040 | -0.038 | 0.14** | 0.035 | 0.056 | 0.041 | 0.019 | -0.011 |
|  | (0.427) | (0.473) | (0.003) | (0.452) | (0.175) | (0.365) | (0.699) | (0.813) |
| [2] Having 2 children | 0.20*** | 0.058 | 0.28*** | 0.12** | 0.029 | 0.014 | 0.13** | 0.060 |
|  | (0.000) | (0.229) | (0.000) | (0.008) | (0.452) | (0.753) | (0.004) | (0.179) |
| [3] Having 3 or more children | 0.12* | -0.016 | 0.22*** | 0.040 | -0.0073 | -0.035 | 0.025 | -0.047 |
|  | (0.019) | (0.757) | (0.000) | (0.414) | (0.866) | (0.457) | (0.617) | (0.344) |
| Number of resident children | -0.0067 | -0.025 | -0.094*** | -0.11*** | -0.020 | -0.024 | -0.017 | -0.030 |
|  | (0.713) | (0.162) | (0.000) | (0.000) | (0.199) | (0.129) | (0.339) | (0.072) |
| Number of grandchildren | -0.0033 | 0.014** | -0.025*** | -0.0020 | 0.013** | 0.016*** | -0.016** | -0.00068 |
|  | (0.528) | (0.007) | (0.000) | (0.672) | (0.002) | (0.000) | (0.003) | (0.889) |
| **Controls** |  |  |  |  |  |  |  |  |
| Age at interview | 0.052** | 0.083*** | 0.14*** | 0.15*** | -0.031* | -0.025 | 0.13*** | 0.12*** |
|  | (0.002) | (0.000) | (0.000) | (0.000) | (0.016) | (0.080) | (0.000) | (0.000) |
| Age at interview, squared | -0.00036** | -0.00044*** | -0.0012*** | -0.0011*** | 0.00022* | 0.00021* | -0.0011*** | -0.00087*** |
|  | (0.003) | (0.000) | (0.000) | (0.000) | (0.019) | (0.048) | (0.000) | (0.000) |
| sh_country==[2]BEL | -0.43*** | -0.43*** | -0.71*** | -0.68*** | -0.54*** | -0.56*** | -0.32*** | -0.25*** |
|  | (0.000) | (0.000) | (0.000) | (0.000) | (0.000) | (0.000) | (0.000) | (0.000) |
| sh_country==[3]CHE | 0.13** | -0.13* | 0.23*** | -0.048 | -0.28*** | -0.39*** | 0.042 | -0.19*** |
|  | (0.007) | (0.010) | (0.000) | (0.325) | (0.000) | (0.000) | (0.419) | (0.000) |
| sh_country==[4]CZE | -0.88*** | -0.53*** | -1.37*** | -0.95*** | -0.32*** | -0.27*** | -0.11* | 0.23*** |
|  | (0.000) | (0.000) | (0.000) | (0.000) | (0.000) | (0.000) | (0.032) | (0.000) |
| sh_country==[5]DEU | -0.54*** | -0.46*** | -0.28*** | -0.19** | -0.41*** | -0.41*** | -0.13 | -0.047 |
|  | (0.000) | (0.000) | (0.000) | (0.003) | (0.000) | (0.000) | (0.063) | (0.478) |
| sh_country==[6]DNK | 0.24*** | -0.038 | 0.20*** | -0.11* | 0.11* | 0.017 | 0.20*** | 0.0077 |
|  | (0.000) | (0.500) | (0.000) | (0.030) | (0.021) | (0.751) | (0.001) | (0.886) |
| sh_country==[7]ESP | -0.61*** | -0.33*** | -0.87*** | -0.42*** | -0.31*** | -0.27*** | -0.23*** | 0.045 |
|  | (0.000) | (0.000) | (0.000) | (0.000) | (0.000) | (0.000) | (0.000) | (0.459) |
| sh_country==[8]EST | -1.65*** | -1.33*** | -1.32*** | -0.90*** | -0.46*** | -0.43*** | -0.88*** | -0.44*** |
|  | (0.000) | (0.000) | (0.000) | (0.000) | (0.000) | (0.000) | (0.000) | (0.000) |
| sh_country==[9]FRA | -0.91*** | -0.79*** | -0.44*** | -0.28*** | -0.46*** | -0.47*** | -0.49*** | -0.31*** |
|  | (0.000) | (0.000) | (0.000) | (0.000) | (0.000) | (0.000) | (0.000) | (0.000) |
| sh_country==[10]HUN | -1.53*** | -1.02*** | -1.29*** | -0.65*** | -0.17** | -0.12* | -0.70*** | -0.21*** |
|  | (0.000) | (0.000) | (0.000) | (0.000) | (0.002) | (0.045) | (0.000) | (0.001) |
| sh_country==[11]ITA | -0.58*** | -0.46*** | -1.46*** | -1.27*** | -0.21*** | -0.19*** | -0.37*** | -0.27*** |
|  | (0.000) | (0.000) | (0.000) | (0.000) | (0.000) | (0.000) | (0.000) | (0.000) |
| sh_country==[12]NLD | -0.22*** | -0.35*** | 0.28*** | 0.16** | -0.55*** | -0.60*** | 0.24*** | 0.13** |
|  | (0.000) | (0.000) | (0.000) | (0.002) | (0.000) | (0.000) | (0.000) | (0.010) |
| sh_country==[13]POL | -0.81*** | -0.33*** | -1.07*** | -0.46*** | -0.27*** | -0.21** | -0.84*** | -0.35*** |
|  | (0.000) | (0.000) | (0.000) | (0.000) | (0.000) | (0.004) | (0.000) | (0.000) |
| sh_country==[14]PRT | -1.05*** | -0.59*** | -2.00*** | -1.30*** | -0.083 | 0.080 | -0.76*** | -0.24** |
|  | (0.000) | (0.000) | (0.000) | (0.000) | (0.133) | (0.208) | (0.000) | (0.001) |
| sh_country==[15]SVN | -0.89*** | -0.63*** | -0.052 | 0.29*** | -0.30*** | -0.29*** | -0.30*** | -0.068 |
|  | (0.000) | (0.000) | (0.393) | (0.000) | (0.000) | (0.000) | (0.000) | (0.253) |
| sh_country==[16]SWE | 0.059 | -0.16* | -0.25*** | -0.43*** | -0.11 | -0.18** | 0.041 | -0.073 |
|  | (0.336) | (0.011) | (0.000) | (0.000) | (0.060) | (0.004) | (0.501) | (0.222) |
| Divorced/living separated |  | -0.0038 |  | 0.027 |  | -0.094 |  | -0.13* |
|  |  | (0.958) |  | (0.663) |  | (0.151) |  | (0.049) |
| Widowed |  | 0.085 |  | 0.11 |  | -0.054 |  | -0.25*** |
|  |  | (0.284) |  | (0.110) |  | (0.466) |  | (0.001) |
| [1] Suburbs of big city |  | 0.010 |  | 0.033 |  | 0.038 |  | -0.066 |
|  |  | (0.817) |  | (0.420) |  | (0.344) |  | (0.114) |
| [2] Large town |  | 0.049 |  | 0.072 |  | 0.11** |  | -0.071 |
|  |  | (0.252) |  | (0.057) |  | (0.003) |  | (0.070) |
| [3] Small town |  | 0.10** |  | 0.11** |  | 0.13*** |  | 0.044 |
|  |  | (0.010) |  | (0.002) |  | (0.000) |  | (0.210) |
| [4] Rural area/village |  | 0.068 |  | 0.10** |  | 0.12*** |  | 0.029 |
|  |  | (0.073) |  | (0.002) |  | (0.000) |  | (0.398) |
| Employment, current job |  | 0.29*** |  | 0.22*** |  | 0.058* |  | 0.13*** |
|  |  | (0.000) |  | (0.000) |  | (0.044) |  | (0.000) |
| Self-employment, current job |  | 0.21*** |  | 0.21*** |  | 0.032 |  | 0.078* |
|  |  | (0.000) |  | (0.000) |  | (0.407) |  | (0.045) |
| [1] Primary school |  | 0.21* |  | 0.39*** |  | 0.019 |  | 0.18* |
|  |  | (0.019) |  | (0.000) |  | (0.798) |  | (0.038) |
| [2] Lower secondary school |  | 0.22* |  | 0.48*** |  | 0.037 |  | 0.26** |
|  |  | (0.019) |  | (0.000) |  | (0.634) |  | (0.003) |
| [3] Upper secondary school |  | 0.24** |  | 0.59*** |  | 0.037 |  | 0.28** |
|  |  | (0.007) |  | (0.000) |  | (0.628) |  | (0.001) |
| [4] Post-secondary non-tertiary education |  | 0.34** |  | 0.62*** |  | 0.070 |  | 0.27** |
|  |  | (0.001) |  | (0.000) |  | (0.421) |  | (0.005) |
| [5] First stage tertiary education |  | 0.29** |  | 0.63*** |  | 0.034 |  | 0.26** |
|  |  | (0.002) |  | (0.000) |  | (0.659) |  | (0.003) |
| [6] Second stage tertiary education |  | 0.41** |  | 0.72*** |  | -0.064 |  | 0.23 |
|  |  | (0.002) |  | (0.000) |  | (0.605) |  | (0.080) |
| [1] Fair |  | 1.05*** |  | 1.12*** |  | 0.11** |  | 1.20*** |
|  |  | (0.000) |  | (0.000) |  | (0.004) |  | (0.000) |
| [2] Good |  | 1.48*** |  | 1.75*** |  | 0.14*** |  | 1.84*** |
|  |  | (0.000) |  | (0.000) |  | (0.000) |  | (0.000) |
| [3] Very good |  | 1.82*** |  | 2.13*** |  | 0.28*** |  | 2.12*** |
|  |  | (0.000) |  | (0.000) |  | (0.000) |  | (0.000) |
| [4] Excellent |  | 2.10*** |  | 2.44*** |  | 0.41*** |  | 2.22*** |
|  |  | (0.000) |  | (0.000) |  | (0.000) |  | (0.000) |
| Drugs for depression |  | -0.44*** |  | -0.58*** |  | -0.078* |  | -1.22*** |
|  |  | (0.000) |  | (0.000) |  | (0.036) |  | (0.000) |
| [1] Middle income |  | 0.22*** |  | 0.26*** |  | 0.037 |  | 0.13*** |
|  |  | (0.000) |  | (0.000) |  | (0.289) |  | (0.000) |
| [2] Upper middle income |  | 0.29*** |  | 0.30*** |  | 0.022 |  | 0.16*** |
|  |  | (0.000) |  | (0.000) |  | (0.522) |  | (0.000) |
| [3] High income |  | 0.26*** |  | 0.30*** |  | 0.057 |  | 0.11*** |
|  |  | (0.000) |  | (0.000) |  | (0.071) |  | (0.001) |
| _cons | 5.42*** | 1.99** | 3.37*** | -0.14 | 7.81*** | 7.14*** | 4.35*** | 2.33*** |
|  | (0.000) | (0.001) | (0.000) | (0.786) | (0.000) | (0.000) | (0.000) | (0.000) |
| N | 22847 | 20648 | 22149 | 20067 | 22962 | 20735 | 22705 | 20518 |
| R² | 0.13 | 0.25 | 0.18 | 0.36 | 0.13 | 0.14 | 0.06 | 0.27 |
| adjusted R² | 0.13 | 0.25 | 0.18 | 0.36 | 0.13 | 0.14 | 0.06 | 0.27 |
