# Supplementary material for: Marriage, parenthood and social network: Subjective well-being and mental health in old age
Source: PLoS One. 2019 Jul 24;14(7):e0218704. doi: 10.1371/journal.pone.0218704 (PMC6656342; doi:10.1371/journal.pone.0218704)
Supplement: S10 Table — (DOCX) [file pone.0218704.s015.docx]

**S10 Table. Regressing well-being and mental health on network types controlling for network size and family status for all countries, female respondents**

|  | Life satisfaction | | Quality of life (CASP-12) | | Network satisfaction | | Lack of depressive symptoms (EURO-D) | |
| --- | --- | --- | --- | --- | --- | --- | --- | --- |
|  | A | B | A | B | A | B | A | B |
| [1] Partner | 0.45*** | 0.50*** | 0.29*** | 0.29*** | 2.38*** | 2.48*** | 0.48*** | 0.46*** |
|  | (0.000) | (0.000) | (0.000) | (0.000) | (0.000) | (0.000) | (0.000) | (0.000) |
| [2] Children | 0.15 | 0.27** | -0.11 | 0.022 | 2.57*** | 2.65*** | 0.041 | 0.22** |
|  | (0.087) | (0.002) | (0.136) | (0.747) | (0.000) | (0.000) | (0.641) | (0.007) |
| [3] Other Relatives | 0.16 | 0.26** | 0.061 | 0.13 | 2.34*** | 2.43*** | 0.020 | 0.12 |
|  | (0.058) | (0.003) | (0.426) | (0.061) | (0.000) | (0.000) | (0.819) | (0.135) |
| [4] Family | 0.25** | 0.36*** | 0.044 | 0.14* | 2.47*** | 2.55*** | 0.16 | 0.30*** |
|  | (0.003) | (0.000) | (0.559) | (0.050) | (0.000) | (0.000) | (0.060) | (0.000) |
| [5] Friends | 0.13 | 0.21* | 0.082 | 0.17* | 2.25*** | 2.34*** | 0.0080 | 0.12 |
|  | (0.133) | (0.013) | (0.279) | (0.016) | (0.000) | (0.000) | (0.928) | (0.128) |
| [6] Diverse | 0.041 | 0.23** | -0.11 | 0.066 | 2.20*** | 2.29*** | -0.15 | 0.063 |
|  | (0.647) | (0.009) | (0.157) | (0.363) | (0.000) | (0.000) | (0.095) | (0.458) |
| Size of social network | 0.11*** | 0.074*** | 0.12*** | 0.077*** | 0.058*** | 0.056*** | 0.059*** | 0.016* |
|  | (0.000) | (0.000) | (0.000) | (0.000) | (0.000) | (0.000) | (0.000) | (0.040) |
| Married/registered partnership | 0.47*** | 0.33*** | 0.22*** | 0.17*** | 0.054** | 0.12* | 0.16*** | -0.034 |
|  | (0.000) | (0.000) | (0.000) | (0.001) | (0.004) | (0.011) | (0.000) | (0.536) |
| [1] Having 1 child | -0.070 | -0.060 | 0.039 | 0.040 | 0.11** | 0.089* | -0.12* | -0.062 |
|  | (0.124) | (0.203) | (0.372) | (0.348) | (0.004) | (0.029) | (0.015) | (0.195) |
| [2] Having 2 children | 0.068 | 0.070 | 0.11** | 0.085* | 0.12*** | 0.10** | 0.027 | 0.065 |
|  | (0.119) | (0.131) | (0.008) | (0.040) | (0.001) | (0.010) | (0.559) | (0.163) |
| [3] Having 3 or more children | -0.0012 | 0.017 | 0.042 | 0.036 | 0.13** | 0.11* | -0.055 | 0.0093 |
|  | (0.980) | (0.742) | (0.368) | (0.435) | (0.001) | (0.015) | (0.306) | (0.859) |
| Number of resident children | -0.048* | -0.075*** | -0.095*** | -0.13*** | -0.020 | -0.025 | -0.010 | -0.021 |
|  | (0.012) | (0.000) | (0.000) | (0.000) | (0.148) | (0.084) | (0.616) | (0.276) |
| Number of grandchildren | -0.0056 | 0.0062 | -0.018*** | 0.00098 | 0.010** | 0.012** | -0.024*** | -0.0052 |
|  | (0.264) | (0.203) | (0.000) | (0.812) | (0.005) | (0.001) | (0.000) | (0.276) |
| **Controls** |  |  |  |  |  |  |  |  |
| Age at interview | -0.0037 | 0.0096 | 0.097*** | 0.11*** | -0.0057 | -0.0085 | 0.11*** | 0.100*** |
|  | (0.792) | (0.526) | (0.000) | (0.000) | (0.586) | (0.467) | (0.000) | (0.000) |
| Age at interview, squared | 0.000064 | 0.000092 | -0.00088*** | -0.00084*** | 0.000024 | 0.000050 | -0.00089*** | -0.00068*** |
|  | (0.535) | (0.401) | (0.000) | (0.000) | (0.758) | (0.554) | (0.000) | (0.000) |
| sh_country==[2]BEL | -0.61*** | -0.45*** | -0.84*** | -0.63*** | -0.70*** | -0.67*** | -0.62*** | -0.31*** |
|  | (0.000) | (0.000) | (0.000) | (0.000) | (0.000) | (0.000) | (0.000) | (0.000) |
| sh_country==[3]CHE | 0.045 | -0.12* | 0.27*** | 0.088 | -0.44*** | -0.42*** | -0.071 | -0.23*** |
|  | (0.326) | (0.011) | (0.000) | (0.050) | (0.000) | (0.000) | (0.157) | (0.000) |
| sh_country==[4]CZE | -0.99*** | -0.65*** | -1.39*** | -0.99*** | -0.41*** | -0.39*** | -0.34*** | 0.036 |
|  | (0.000) | (0.000) | (0.000) | (0.000) | (0.000) | (0.000) | (0.000) | (0.467) |
| sh_country==[5]DEU | -0.57*** | -0.47*** | -0.24*** | -0.10 | -0.47*** | -0.44*** | -0.39*** | -0.25*** |
|  | (0.000) | (0.000) | (0.000) | (0.087) | (0.000) | (0.000) | (0.000) | (0.000) |
| sh_country==[6]DNK | 0.30*** | 0.094 | 0.29*** | 0.057 | -0.023 | -0.030 | 0.11 | -0.076 |
|  | (0.000) | (0.082) | (0.000) | (0.243) | (0.581) | (0.492) | (0.067) | (0.193) |
| sh_country==[7]ESP | -0.86*** | -0.42*** | -1.24*** | -0.65*** | -0.35*** | -0.31*** | -1.08*** | -0.45*** |
|  | (0.000) | (0.000) | (0.000) | (0.000) | (0.000) | (0.000) | (0.000) | (0.000) |
| sh_country==[8]EST | -1.49*** | -1.13*** | -1.11*** | -0.68*** | -0.41*** | -0.30*** | -1.00*** | -0.46*** |
|  | (0.000) | (0.000) | (0.000) | (0.000) | (0.000) | (0.000) | (0.000) | (0.000) |
| sh_country==[9]FRA | -1.03*** | -0.84*** | -0.59*** | -0.30*** | -0.55*** | -0.52*** | -0.80*** | -0.45*** |
|  | (0.000) | (0.000) | (0.000) | (0.000) | (0.000) | (0.000) | (0.000) | (0.000) |
| sh_country==[10]HUN | -1.62*** | -1.02*** | -1.37*** | -0.67*** | -0.20*** | -0.11* | -1.11*** | -0.43*** |
|  | (0.000) | (0.000) | (0.000) | (0.000) | (0.000) | (0.019) | (0.000) | (0.000) |
| sh_country==[11]ITA | -0.74*** | -0.48*** | -1.71*** | -1.40*** | -0.26*** | -0.23*** | -0.74*** | -0.40*** |
|  | (0.000) | (0.000) | (0.000) | (0.000) | (0.000) | (0.000) | (0.000) | (0.000) |
| sh_country==[12]NLD | -0.34*** | -0.35*** | 0.22*** | 0.23*** | -0.71*** | -0.72*** | -0.059 | 0.015 |
|  | (0.000) | (0.000) | (0.000) | (0.000) | (0.000) | (0.000) | (0.303) | (0.777) |
| sh_country==[13]POL | -0.95*** | -0.37*** | -1.18*** | -0.53*** | -0.23*** | -0.15* | -1.21*** | -0.58*** |
|  | (0.000) | (0.000) | (0.000) | (0.000) | (0.000) | (0.015) | (0.000) | (0.000) |
| sh_country==[14]PRT | -1.51*** | -0.72*** | -2.25*** | -1.34*** | -0.16*** | 0.012 | -1.54*** | -0.50*** |
|  | (0.000) | (0.000) | (0.000) | (0.000) | (0.001) | (0.819) | (0.000) | (0.000) |
| sh_country==[15]SVN | -0.77*** | -0.49*** | -0.13* | 0.25*** | -0.30*** | -0.22*** | -0.46*** | -0.17** |
|  | (0.000) | (0.000) | (0.019) | (0.000) | (0.000) | (0.000) | (0.000) | (0.004) |
| sh_country==[16]SWE | 0.024 | -0.038 | -0.12* | -0.18*** | -0.16*** | -0.18*** | 0.0013 | -0.0066 |
|  | (0.678) | (0.511) | (0.025) | (0.001) | (0.001) | (0.000) | (0.983) | (0.911) |
| Divorced/living separated |  | -0.18** |  | -0.10 |  | 0.014 |  | -0.13* |
|  |  | (0.003) |  | (0.058) |  | (0.786) |  | (0.032) |
| Widowed |  | 0.023 |  | 0.12* |  | 0.12* |  | -0.12* |
|  |  | (0.705) |  | (0.025) |  | (0.020) |  | (0.037) |
| [1] Suburbs of big city |  | 0.010 |  | 0.028 |  | 0.00070 |  | -0.11* |
|  |  | (0.802) |  | (0.443) |  | (0.983) |  | (0.013) |
| [2] Large town |  | 0.043 |  | 0.0086 |  | 0.048 |  | -0.078* |
|  |  | (0.256) |  | (0.798) |  | (0.109) |  | (0.042) |
| [3] Small town |  | 0.11** |  | 0.052 |  | 0.057* |  | -0.0069 |
|  |  | (0.002) |  | (0.097) |  | (0.040) |  | (0.848) |
| [4] Rural area/village |  | 0.068 |  | 0.033 |  | -0.029 |  | -0.032 |
|  |  | (0.053) |  | (0.275) |  | (0.279) |  | (0.359) |
| Employment, current job |  | 0.075* |  | 0.13*** |  | -0.0089 |  | 0.063* |
|  |  | (0.017) |  | (0.000) |  | (0.709) |  | (0.048) |
| Self-employment, current job |  | 0.084 |  | 0.082 |  | -0.098* |  | 0.077 |
|  |  | (0.111) |  | (0.092) |  | (0.034) |  | (0.175) |
| [1] Primary school |  | 0.0061 |  | 0.32*** |  | -0.061 |  | 0.25*** |
|  |  | (0.935) |  | (0.000) |  | (0.275) |  | (0.001) |
| [2] Lower secondary school |  | 0.039 |  | 0.39*** |  | -0.097 |  | 0.33*** |
|  |  | (0.609) |  | (0.000) |  | (0.095) |  | (0.000) |
| [3] Upper secondary school |  | 0.076 |  | 0.49*** |  | -0.091 |  | 0.48*** |
|  |  | (0.321) |  | (0.000) |  | (0.118) |  | (0.000) |
| [4] Post-secondary non-tertiary education |  | 0.12 |  | 0.64*** |  | -0.078 |  | 0.62*** |
|  |  | (0.189) |  | (0.000) |  | (0.260) |  | (0.000) |
| [5] First stage tertiary education |  | 0.17* |  | 0.53*** |  | -0.15* |  | 0.51*** |
|  |  | (0.025) |  | (0.000) |  | (0.012) |  | (0.000) |
| [6] Second stage tertiary education |  | 0.42** |  | 0.71*** |  | 0.065 |  | 0.61*** |
|  |  | (0.003) |  | (0.000) |  | (0.558) |  | (0.000) |
| [1] Fair |  | 1.00*** |  | 1.10*** |  | 0.15*** |  | 1.27*** |
|  |  | (0.000) |  | (0.000) |  | (0.000) |  | (0.000) |
| [2] Good |  | 1.53*** |  | 1.80*** |  | 0.19*** |  | 2.03*** |
|  |  | (0.000) |  | (0.000) |  | (0.000) |  | (0.000) |
| [3] Very good |  | 1.86*** |  | 2.19*** |  | 0.31*** |  | 2.40*** |
|  |  | (0.000) |  | (0.000) |  | (0.000) |  | (0.000) |
| [4] Excellent |  | 2.22*** |  | 2.51*** |  | 0.47*** |  | 2.54*** |
|  |  | (0.000) |  | (0.000) |  | (0.000) |  | (0.000) |
| Drugs for depression |  | -0.51*** |  | -0.63*** |  | -0.11*** |  | -1.16*** |
|  |  | (0.000) |  | (0.000) |  | (0.000) |  | (0.000) |
| [1] Middle income |  | 0.095** |  | 0.13*** |  | -0.026 |  | 0.051 |
|  |  | (0.007) |  | (0.000) |  | (0.362) |  | (0.170) |
| [2] Upper middle income |  | 0.18*** |  | 0.13*** |  | 0.011 |  | 0.010 |
|  |  | (0.000) |  | (0.000) |  | (0.690) |  | (0.770) |
| [3] High income |  | 0.22*** |  | 0.20*** |  | -0.036 |  | 0.041 |
|  |  | (0.000) |  | (0.000) |  | (0.174) |  | (0.225) |
| _cons | 7.46*** | 4.85*** | 4.65*** | 1.25** | 6.88*** | 6.70*** | 4.91*** | 2.21*** |
|  | (0.000) | (0.000) | (0.000) | (0.006) | (0.000) | (0.000) | (0.000) | (0.000) |
| N | 29401 | 26321 | 28363 | 25472 | 29551 | 26426 | 29236 | 26172 |
| R² | 0.13 | 0.25 | 0.21 | 0.38 | 0.12 | 0.13 | 0.081 | 0.3 |
| adjusted R² | 0.13 | 0.25 | 0.21 | 0.38 | 0.12 | 0.13 | 0.08 | 0.29 |
