# Supplementary material for: Marriage, parenthood and social network: Subjective well-being and mental health in old age
Source: PLoS One. 2019 Jul 24;14(7):e0218704. doi: 10.1371/journal.pone.0218704 (PMC6656342; doi:10.1371/journal.pone.0218704)
Supplement: S11 Table — (DOCX) [file pone.0218704.s016.docx]

**S11 Table. Well-being and mental health measures conditional on network size over all countries**

|  | Life satisfaction | | | Quality of life (CASP-12) | | | Network satisfaction | | | Lack of depressive symptoms (EURO-D) | | |
| --- | --- | --- | --- | --- | --- | --- | --- | --- | --- | --- | --- | --- |
| Size | All (1) | Male (2) | Female (3) | All (1) | Male (2) | Female (3) | All (1) | Male (2) | Female (3) | All (1) | Male (2) | Female (3) |
| 0 | 6.93 | 6.98 | 6.88 | 6.48 | 6.67 | 6.29 | 6.49 | 6.60 | 6.38 | 7.59 | 7.99 | 7.18 |
| 1 | 7.45 | 7.58 | 7.32 | 6.80 | 6.97 | 6.61 | 8.86 | 8.89 | 8.83 | 7.89 | 8.25 | 7.49 |
| 2 | 7.48 | 7.61 | 7.38 | 6.84 | 7.03 | 6.69 | 8.90 | 8.83 | 8.95 | 7.81 | 8.26 | 7.46 |
| 3 | 7.62 | 7.75 | 7.54 | 7.07 | 7.21 | 6.97 | 8.95 | 8.86 | 9.02 | 7.83 | 8.26 | 7.56 |
| 4 | 7.76 | 7.83 | 7.72 | 7.18 | 7.27 | 7.12 | 8.95 | 8.82 | 9.03 | 7.90 | 8.34 | 7.63 |
| 5 | 7.82 | 7.92 | 7.76 | 7.34 | 7.50 | 7.26 | 8.96 | 8.87 | 9.00 | 7.92 | 8.43 | 7.66 |
| 6 | 7.99 | 8.06 | 7.96 | 7.45 | 7.55 | 7.39 | 8.96 | 8.82 | 9.03 | 8.01 | 8.46 | 7.80 |
| 7 | 8.04 | 8.06 | 8.03 | 7.46 | 7.44 | 7.47 | 8.99 | 8.84 | 9.07 | 8.01 | 8.35 | 7.83 |

For each well-being and mental health measure column (1)-(3) represent the average conditional on the network size for all respondents and by gender.
