# Supplementary material for: Marriage, parenthood and social network: Subjective well-being and mental health in old age
Source: PLoS One. 2019 Jul 24;14(7):e0218704. doi: 10.1371/journal.pone.0218704 (PMC6656342; doi:10.1371/journal.pone.0218704)
Supplement: S12 Table — (DOCX) [file pone.0218704.s017.docx]

**S12 Table. Regressing well-being and mental health on network types controlling for network size, relational dynamics and family status for all countries, all respondents with social support network**

|  | Life satisfaction | | Quality of life (CASP-12) | | Network satisfaction | | Lack of depressive symptoms (EURO-D) | |
| --- | --- | --- | --- | --- | --- | --- | --- | --- |
|  | A | B | A | B | A | B | A | B |
| [2] Children | -0.22*** | -0.12*** | -0.29*** | -0.15*** | 0.14*** | 0.12*** | -0.28*** | -0.084* |
|  | (0.000) | (0.000) | (0.000) | (0.000) | (0.000) | (0.000) | (0.000) | (0.011) |
| [3] Other Relatives | -0.12*** | -0.059 | -0.053 | 0.025 | 0.097*** | 0.092*** | -0.25*** | -0.14*** |
|  | (0.000) | (0.072) | (0.093) | (0.400) | (0.000) | (0.000) | (0.000) | (0.000) |
| [4] Family | -0.10*** | -0.023 | -0.14*** | -0.039 | 0.076*** | 0.068*** | -0.16*** | -0.022 |
|  | (0.000) | (0.403) | (0.000) | (0.115) | (0.000) | (0.001) | (0.000) | (0.422) |
| [5] Friends | -0.052 | -0.029 | 0.057 | 0.11*** | 0.13*** | 0.12*** | -0.21*** | -0.12*** |
|  | (0.101) | (0.352) | (0.058) | (0.000) | (0.000) | (0.000) | (0.000) | (0.000) |
| [6] Diverse | -0.15*** | -0.055 | -0.13*** | -0.011 | 0.043 | 0.032 | -0.34*** | -0.18*** |
|  | (0.000) | (0.109) | (0.000) | (0.713) | (0.085) | (0.220) | (0.000) | (0.000) |
| Size of social network | 0.099*** | 0.074*** | 0.11*** | 0.074*** | 0.073*** | 0.072*** | 0.052*** | 0.024*** |
|  | (0.000) | (0.000) | (0.000) | (0.000) | (0.000) | (0.000) | (0.000) | (0.000) |
| Average contact 0-6 | 0.064*** | 0.066*** | 0.068*** | 0.066*** | 0.18*** | 0.17*** | 0.032** | 0.030** |
|  | (0.000) | (0.000) | (0.000) | (0.000) | (0.000) | (0.000) | (0.009) | (0.008) |
| Average closeness 0-3 | 0.32*** | 0.26*** | 0.30*** | 0.24*** | 0.70*** | 0.68*** | 0.17*** | 0.11*** |
|  | (0.000) | (0.000) | (0.000) | (0.000) | (0.000) | (0.000) | (0.000) | (0.000) |
| Average proximity 0-5 | -0.041*** | -0.0045 | -0.076*** | -0.030*** | -0.052*** | -0.053*** | -0.033** | 0.019 |
|  | (0.000) | (0.657) | (0.000) | (0.001) | (0.000) | (0.000) | (0.002) | (0.063) |
| Married/registered partnership | 0.44*** | 0.33*** | 0.21*** | 0.14*** | -0.013 | 0.027 | 0.18*** | -0.042 |
|  | (0.000) | (0.000) | (0.000) | (0.000) | (0.338) | (0.385) | (0.000) | (0.290) |
| [1] Having 1 child | -0.050 | -0.083* | 0.056 | 0.0039 | 0.023 | -0.00071 | -0.069* | -0.065 |
|  | (0.140) | (0.018) | (0.082) | (0.901) | (0.332) | (0.978) | (0.049) | (0.060) |
| [2] Having 2 children | 0.098** | 0.035 | 0.15*** | 0.064* | 0.028 | 0.0041 | 0.058 | 0.037 |
|  | (0.002) | (0.291) | (0.000) | (0.036) | (0.216) | (0.872) | (0.083) | (0.264) |
| [3] Having 3 or more children | 0.028 | -0.019 | 0.086* | 0.0080 | 0.019 | -0.0071 | -0.034 | -0.038 |
|  | (0.438) | (0.610) | (0.013) | (0.811) | (0.439) | (0.797) | (0.368) | (0.294) |
| Number of resident children | -0.030* | -0.058*** | -0.089*** | -0.12*** | -0.027** | -0.030** | -0.010 | -0.030* |
|  | (0.025) | (0.000) | (0.000) | (0.000) | (0.003) | (0.002) | (0.459) | (0.024) |
| Number of grandchildren | -0.0072 | 0.0064 | -0.021*** | -0.0016 | 0.0095*** | 0.010*** | -0.021*** | -0.0046 |
|  | (0.052) | (0.077) | (0.000) | (0.600) | (0.000) | (0.000) | (0.000) | (0.188) |
| **Controls** |  |  |  |  |  |  |  |  |
| Female | -0.074*** | 0.016 | -0.19*** | -0.083*** | 0.055*** | 0.051*** | -0.65*** | -0.51*** |
|  | (0.000) | (0.319) | (0.000) | (0.000) | (0.000) | (0.000) | (0.000) | (0.000) |
| Age at interview | 0.028* | 0.047*** | 0.12*** | 0.13*** | 0.0039 | 0.0032 | 0.12*** | 0.11*** |
|  | (0.010) | (0.000) | (0.000) | (0.000) | (0.583) | (0.686) | (0.000) | (0.000) |
| Age at interview, squared | -0.00016* | -0.00018* | -0.0011*** | -0.00100*** | -0.000013 | -0.0000018 | -0.0010*** | -0.00079*** |
|  | (0.047) | (0.032) | (0.000) | (0.000) | (0.806) | (0.975) | (0.000) | (0.000) |
| sh_country==[2]BEL | -0.37*** | -0.31*** | -0.62*** | -0.52*** | -0.27*** | -0.28*** | -0.40*** | -0.23*** |
|  | (0.000) | (0.000) | (0.000) | (0.000) | (0.000) | (0.000) | (0.000) | (0.000) |
| sh_country==[3]CHE | 0.35*** | 0.10** | 0.51*** | 0.25*** | 0.21*** | 0.20*** | 0.100** | -0.12** |
|  | (0.000) | (0.006) | (0.000) | (0.000) | (0.000) | (0.000) | (0.009) | (0.001) |
| sh_country==[4]CZE | -0.80*** | -0.50*** | -1.25*** | -0.88*** | -0.044 | -0.046 | -0.16*** | 0.17*** |
|  | (0.000) | (0.000) | (0.000) | (0.000) | (0.092) | (0.121) | (0.000) | (0.000) |
| sh_country==[5]DEU | -0.33*** | -0.27*** | -0.036 | 0.038 | 0.067 | 0.067 | -0.15** | -0.076 |
|  | (0.000) | (0.000) | (0.464) | (0.405) | (0.076) | (0.093) | (0.003) | (0.114) |
| sh_country==[6]DNK | 0.44*** | 0.18*** | 0.39*** | 0.11** | 0.36*** | 0.32*** | 0.22*** | 0.017 |
|  | (0.000) | (0.000) | (0.000) | (0.002) | (0.000) | (0.000) | (0.000) | (0.665) |
| sh_country==[7]ESP | -0.66*** | -0.33*** | -0.99*** | -0.49*** | -0.17*** | -0.16*** | -0.66*** | -0.21*** |
|  | (0.000) | (0.000) | (0.000) | (0.000) | (0.000) | (0.000) | (0.000) | (0.000) |
| sh_country==[8]EST | -1.30*** | -1.01*** | -0.96*** | -0.59*** | 0.16*** | 0.20*** | -0.81*** | -0.34*** |
|  | (0.000) | (0.000) | (0.000) | (0.000) | (0.000) | (0.000) | (0.000) | (0.000) |
| sh_country==[9]FRA | -0.80*** | -0.66*** | -0.36*** | -0.16*** | -0.11*** | -0.12*** | -0.58*** | -0.32*** |
|  | (0.000) | (0.000) | (0.000) | (0.000) | (0.000) | (0.000) | (0.000) | (0.000) |
| sh_country==[10]HUN | -1.49*** | -0.97*** | -1.25*** | -0.61*** | 0.0041 | 0.026 | -0.88*** | -0.33*** |
|  | (0.000) | (0.000) | (0.000) | (0.000) | (0.890) | (0.429) | (0.000) | (0.000) |
| sh_country==[11]ITA | -0.54*** | -0.39*** | -1.48*** | -1.27*** | 0.0061 | -0.0024 | -0.52*** | -0.33*** |
|  | (0.000) | (0.000) | (0.000) | (0.000) | (0.819) | (0.931) | (0.000) | (0.000) |
| sh_country==[12]NLD | -0.21*** | -0.28*** | 0.32*** | 0.26*** | -0.45*** | -0.49*** | 0.12** | 0.100** |
|  | (0.000) | (0.000) | (0.000) | (0.000) | (0.000) | (0.000) | (0.003) | (0.009) |
| sh_country==[13]POL | -0.72*** | -0.23*** | -0.95*** | -0.37*** | 0.15*** | 0.18*** | -0.96*** | -0.44*** |
|  | (0.000) | (0.000) | (0.000) | (0.000) | (0.000) | (0.000) | (0.000) | (0.000) |
| sh_country==[14]PRT | -1.19*** | -0.58*** | -2.04*** | -1.26*** | 0.10** | 0.23*** | -1.14*** | -0.35*** |
|  | (0.000) | (0.000) | (0.000) | (0.000) | (0.002) | (0.000) | (0.000) | (0.000) |
| sh_country==[15]SVN | -0.67*** | -0.43*** | 0.066 | 0.38*** | 0.064* | 0.074* | -0.30*** | -0.069 |
|  | (0.000) | (0.000) | (0.114) | (0.000) | (0.047) | (0.034) | (0.000) | (0.120) |
| sh_country==[16]SWE | 0.24*** | 0.079 | 0.012 | -0.13** | 0.31*** | 0.26*** | 0.12** | 0.055 |
|  | (0.000) | (0.065) | (0.770) | (0.002) | (0.000) | (0.000) | (0.009) | (0.209) |
| Divorced/living separated |  | -0.11* |  | -0.047 |  | -0.014 |  | -0.12** |
|  |  | (0.026) |  | (0.257) |  | (0.695) |  | (0.006) |
| Widowed |  | 0.075 |  | 0.13** |  | 0.10** |  | -0.15*** |
|  |  | (0.112) |  | (0.002) |  | (0.004) |  | (0.001) |
| [1] Suburbs of big city |  | -0.0012 |  | 0.031 |  | 0.021 |  | -0.090** |
|  |  | (0.968) |  | (0.256) |  | (0.365) |  | (0.003) |
| [2] Large town |  | 0.023 |  | 0.031 |  | 0.073*** |  | -0.092*** |
|  |  | (0.413) |  | (0.223) |  | (0.001) |  | (0.001) |
| [3] Small town |  | 0.089*** |  | 0.068** |  | 0.062** |  | 0.0053 |
|  |  | (0.001) |  | (0.004) |  | (0.001) |  | (0.838) |
| [4] Rural area/village |  | 0.059* |  | 0.072** |  | 0.044* |  | -0.0075 |
|  |  | (0.021) |  | (0.002) |  | (0.020) |  | (0.764) |
| Employment, current job |  | 0.17*** |  | 0.16*** |  | 0.011 |  | 0.099*** |
|  |  | (0.000) |  | (0.000) |  | (0.514) |  | (0.000) |
| Self-employment, current job |  | 0.15*** |  | 0.16*** |  | -0.033 |  | 0.077* |
|  |  | (0.000) |  | (0.000) |  | (0.224) |  | (0.018) |
| [1] Primary school |  | 0.10 |  | 0.37*** |  | -0.051 |  | 0.24*** |
|  |  | (0.092) |  | (0.000) |  | (0.192) |  | (0.000) |
| [2] Lower secondary school |  | 0.14* |  | 0.47*** |  | -0.049 |  | 0.33*** |
|  |  | (0.022) |  | (0.000) |  | (0.223) |  | (0.000) |
| [3] Upper secondary school |  | 0.16** |  | 0.56*** |  | -0.067 |  | 0.43*** |
|  |  | (0.009) |  | (0.000) |  | (0.094) |  | (0.000) |
| [4] Post-secondary non-tertiary education |  | 0.23** |  | 0.66*** |  | -0.041 |  | 0.50*** |
|  |  | (0.001) |  | (0.000) |  | (0.386) |  | (0.000) |
| [5] First stage tertiary education |  | 0.23*** |  | 0.59*** |  | -0.085* |  | 0.43*** |
|  |  | (0.000) |  | (0.000) |  | (0.040) |  | (0.000) |
| [6] Second stage tertiary education |  | 0.37*** |  | 0.70*** |  | -0.091 |  | 0.41*** |
|  |  | (0.000) |  | (0.000) |  | (0.197) |  | (0.000) |
| [1] Fair |  | 1.01*** |  | 1.11*** |  | 0.11*** |  | 1.24*** |
|  |  | (0.000) |  | (0.000) |  | (0.000) |  | (0.000) |
| [2] Good |  | 1.49*** |  | 1.77*** |  | 0.12*** |  | 1.95*** |
|  |  | (0.000) |  | (0.000) |  | (0.000) |  | (0.000) |
| [3] Very good |  | 1.81*** |  | 2.13*** |  | 0.20*** |  | 2.26*** |
|  |  | (0.000) |  | (0.000) |  | (0.000) |  | (0.000) |
| [4] Excellent |  | 2.12*** |  | 2.44*** |  | 0.32*** |  | 2.38*** |
|  |  | (0.000) |  | (0.000) |  | (0.000) |  | (0.000) |
| Drugs for depression |  | -0.49*** |  | -0.61*** |  | -0.090*** |  | -1.19*** |
|  |  | (0.000) |  | (0.000) |  | (0.000) |  | (0.000) |
| [1] Middle income |  | 0.15*** |  | 0.19*** |  | 0.0018 |  | 0.082** |
|  |  | (0.000) |  | (0.000) |  | (0.927) |  | (0.002) |
| [2] Upper middle income |  | 0.23*** |  | 0.21*** |  | 0.010 |  | 0.068** |
|  |  | (0.000) |  | (0.000) |  | (0.598) |  | (0.008) |
| [3] High income |  | 0.23*** |  | 0.23*** |  | -0.022 |  | 0.059* |
|  |  | (0.000) |  | (0.000) |  | (0.215) |  | (0.016) |
| _cons | 5.71*** | 2.78*** | 3.19*** | -0.26 | 6.12*** | 6.05*** | 4.62*** | 2.12*** |
|  | (0.000) | (0.000) | (0.000) | (0.457) | (0.000) | (0.000) | (0.000) | (0.000) |
| N | 50624 | 45591 | 48957 | 44214 | 50869 | 45770 | 50326 | 45316 |
| R² | 0.14 | 0.25 | 0.21 | 0.38 | 0.16 | 0.17 | 0.11 | 0.31 |
| adjusted R² | 0.14 | 0.25 | 0.21 | 0.38 | 0.16 | 0.17 | 0.11 | 0.31 |
