# Supplementary material for: Marriage, parenthood and social network: Subjective well-being and mental health in old age
Source: PLoS One. 2019 Jul 24;14(7):e0218704. doi: 10.1371/journal.pone.0218704 (PMC6656342; doi:10.1371/journal.pone.0218704)
Supplement: S13 Table — (DOCX) [file pone.0218704.s018.docx]

**S13 Table. Regressing well-being and mental health on network types controlling for network size, relational dynamics and family status for all countries, male respondents with social support network**

|  | Life satisfaction | | Quality of life (CASP-12) | | Network satisfaction | | Lack of depressive symptoms (EURO-D) | |
| --- | --- | --- | --- | --- | --- | --- | --- | --- |
|  | A | B | A | B | A | B | A | B |
| [2] Children | -0.11* | -0.049 | -0.12* | -0.014 | 0.15*** | 0.15*** | -0.10* | 0.041 |
|  | (0.029) | (0.330) | (0.015) | (0.762) | (0.000) | (0.000) | (0.043) | (0.399) |
| [3] Other Relatives | -0.083 | -0.039 | 0.023 | 0.084* | 0.044 | 0.048 | -0.11* | -0.040 |
|  | (0.090) | (0.405) | (0.621) | (0.049) | (0.222) | (0.207) | (0.026) | (0.370) |
| [4] Family | -0.055 | 0.013 | -0.086* | 0.0013 | 0.028 | 0.026 | -0.074 | 0.030 |
|  | (0.161) | (0.734) | (0.020) | (0.969) | (0.297) | (0.356) | (0.052) | (0.400) |
| [5] Friends | 0.038 | 0.038 | 0.15*** | 0.16*** | 0.050 | 0.057 | -0.063 | -0.033 |
|  | (0.411) | (0.395) | (0.000) | (0.000) | (0.158) | (0.123) | (0.164) | (0.428) |
| [6] Diverse | -0.042 | -0.033 | 0.0076 | 0.048 | -0.045 | -0.061 | -0.12* | -0.059 |
|  | (0.406) | (0.508) | (0.876) | (0.289) | (0.249) | (0.131) | (0.023) | (0.212) |
| Size of social network | 0.077*** | 0.059*** | 0.084*** | 0.059*** | 0.064*** | 0.065*** | 0.041*** | 0.022* |
|  | (0.000) | (0.000) | (0.000) | (0.000) | (0.000) | (0.000) | (0.000) | (0.018) |
| Average contact 0-6 | 0.055** | 0.053** | 0.052** | 0.051*** | 0.17*** | 0.16*** | 0.031 | 0.021 |
|  | (0.001) | (0.002) | (0.002) | (0.001) | (0.000) | (0.000) | (0.075) | (0.196) |
| Average closeness 0-3 | 0.30*** | 0.23*** | 0.30*** | 0.23*** | 0.70*** | 0.69*** | 0.17*** | 0.11*** |
|  | (0.000) | (0.000) | (0.000) | (0.000) | (0.000) | (0.000) | (0.000) | (0.000) |
| Average proximity 0-5 | -0.045** | -0.0031 | -0.076*** | -0.028 | -0.048*** | -0.045*** | -0.024 | 0.025 |
|  | (0.006) | (0.848) | (0.000) | (0.051) | (0.000) | (0.000) | (0.131) | (0.102) |
| Married/registered partnership | 0.44*** | 0.37*** | 0.22*** | 0.14* | 0.063** | 0.043 | 0.22*** | 0.0011 |
|  | (0.000) | (0.000) | (0.000) | (0.016) | (0.009) | (0.385) | (0.000) | (0.984) |
| [1] Having 1 child | 0.0044 | -0.065 | 0.098* | -0.0076 | -0.018 | -0.036 | -0.0053 | -0.042 |
|  | (0.931) | (0.210) | (0.042) | (0.872) | (0.608) | (0.357) | (0.915) | (0.388) |
| [2] Having 2 children | 0.16*** | 0.030 | 0.22*** | 0.068 | -0.035 | -0.049 | 0.10* | 0.030 |
|  | (0.001) | (0.530) | (0.000) | (0.126) | (0.296) | (0.186) | (0.025) | (0.503) |
| [3] Having 3 or more children | 0.092 | -0.033 | 0.17*** | 0.0011 | -0.046 | -0.068 | 0.0093 | -0.064 |
|  | (0.076) | (0.538) | (0.001) | (0.982) | (0.218) | (0.099) | (0.856) | (0.198) |
| Number of resident children | -0.010 | -0.030 | -0.090*** | -0.11*** | -0.033* | -0.038** | -0.014 | -0.033 |
|  | (0.577) | (0.097) | (0.000) | (0.000) | (0.015) | (0.007) | (0.432) | (0.055) |
| Number of grandchildren | -0.0056 | 0.011* | -0.024*** | -0.0025 | 0.0086* | 0.010* | -0.015** | -0.0014 |
|  | (0.304) | (0.032) | (0.000) | (0.602) | (0.023) | (0.011) | (0.003) | (0.773) |
| **Controls** |  |  |  |  |  |  |  |  |
| Age at interview | 0.057*** | 0.084*** | 0.15*** | 0.16*** | -0.016 | -0.015 | 0.14*** | 0.12*** |
|  | (0.001) | (0.000) | (0.000) | (0.000) | (0.165) | (0.236) | (0.000) | (0.000) |
| Age at interview, squared | -0.00039** | -0.00044*** | -0.0012*** | -0.0012*** | 0.00013 | 0.00014 | -0.0012*** | -0.00090*** |
|  | (0.001) | (0.000) | (0.000) | (0.000) | (0.109) | (0.119) | (0.000) | (0.000) |
| sh_country==[2]BEL | -0.29*** | -0.32*** | -0.58*** | -0.57*** | -0.23*** | -0.26*** | -0.25*** | -0.20*** |
|  | (0.000) | (0.000) | (0.000) | (0.000) | (0.000) | (0.000) | (0.000) | (0.000) |
| sh_country==[3]CHE | 0.35*** | 0.055 | 0.46*** | 0.14** | 0.26*** | 0.19*** | 0.15** | -0.11* |
|  | (0.000) | (0.310) | (0.000) | (0.006) | (0.000) | (0.000) | (0.005) | (0.041) |
| sh_country==[4]CZE | -0.77*** | -0.45*** | -1.26*** | -0.89*** | -0.023 | -0.0063 | -0.053 | 0.27*** |
|  | (0.000) | (0.000) | (0.000) | (0.000) | (0.561) | (0.891) | (0.316) | (0.000) |
| sh_country==[5]DEU | -0.34*** | -0.29*** | -0.076 | -0.029 | 0.052 | 0.046 | -0.0020 | 0.049 |
|  | (0.000) | (0.000) | (0.294) | (0.666) | (0.364) | (0.442) | (0.978) | (0.460) |
| sh_country==[6]DNK | 0.36*** | 0.074 | 0.31*** | -0.013 | 0.36*** | 0.29*** | 0.26*** | 0.047 |
|  | (0.000) | (0.189) | (0.000) | (0.793) | (0.000) | (0.000) | (0.000) | (0.378) |
| sh_country==[7]ESP | -0.52*** | -0.27*** | -0.78*** | -0.37*** | -0.14** | -0.12* | -0.18** | 0.069 |
|  | (0.000) | (0.000) | (0.000) | (0.000) | (0.001) | (0.016) | (0.003) | (0.252) |
| sh_country==[8]EST | -1.42*** | -1.15*** | -1.09*** | -0.72*** | 0.13** | 0.13** | -0.75*** | -0.35*** |
|  | (0.000) | (0.000) | (0.000) | (0.000) | (0.001) | (0.005) | (0.000) | (0.000) |
| sh_country==[9]FRA | -0.75*** | -0.65*** | -0.28*** | -0.16** | -0.089* | -0.10* | -0.41*** | -0.26*** |
|  | (0.000) | (0.000) | (0.000) | (0.001) | (0.027) | (0.017) | (0.000) | (0.000) |
| sh_country==[10]HUN | -1.45*** | -0.97*** | -1.21*** | -0.61*** | -0.023 | -0.0067 | -0.66*** | -0.18** |
|  | (0.000) | (0.000) | (0.000) | (0.000) | (0.621) | (0.901) | (0.000) | (0.004) |
| sh_country==[11]ITA | -0.47*** | -0.41*** | -1.36*** | -1.23*** | 0.032 | 0.025 | -0.34*** | -0.27*** |
|  | (0.000) | (0.000) | (0.000) | (0.000) | (0.419) | (0.557) | (0.000) | (0.000) |
| sh_country==[12]NLD | -0.17*** | -0.30*** | 0.32*** | 0.19*** | -0.41*** | -0.45*** | 0.27*** | 0.16** |
|  | (0.000) | (0.000) | (0.000) | (0.000) | (0.000) | (0.000) | (0.000) | (0.003) |
| sh_country==[13]POL | -0.64*** | -0.19* | -0.90*** | -0.34*** | 0.15** | 0.18** | -0.75*** | -0.29*** |
|  | (0.000) | (0.027) | (0.000) | (0.000) | (0.009) | (0.004) | (0.000) | (0.000) |
| sh_country==[14]PRT | -0.93*** | -0.50*** | -1.92*** | -1.24*** | 0.14** | 0.28*** | -0.73*** | -0.21** |
|  | (0.000) | (0.000) | (0.000) | (0.000) | (0.007) | (0.000) | (0.000) | (0.006) |
| sh_country==[15]SVN | -0.75*** | -0.51*** | 0.081 | 0.39*** | 0.070 | 0.063 | -0.22*** | -0.0019 |
|  | (0.000) | (0.000) | (0.201) | (0.000) | (0.154) | (0.252) | (0.000) | (0.975) |
| sh_country==[16]SWE | 0.22*** | -0.012 | -0.094 | -0.29*** | 0.32*** | 0.26*** | 0.13* | 0.014 |
|  | (0.000) | (0.848) | (0.132) | (0.000) | (0.000) | (0.000) | (0.035) | (0.814) |
| Divorced/living separated |  | -0.0057 |  | 0.033 |  | -0.054 |  | -0.100 |
|  |  | (0.938) |  | (0.605) |  | (0.342) |  | (0.128) |
| Widowed |  | 0.094 |  | 0.12 |  | 0.020 |  | -0.25** |
|  |  | (0.242) |  | (0.078) |  | (0.750) |  | (0.001) |
| [1] Suburbs of big city |  | -0.00046 |  | 0.034 |  | 0.033 |  | -0.069 |
|  |  | (0.992) |  | (0.401) |  | (0.368) |  | (0.102) |
| [2] Large town |  | 0.025 |  | 0.060 |  | 0.086* |  | -0.096* |
|  |  | (0.553) |  | (0.115) |  | (0.011) |  | (0.015) |
| [3] Small town |  | 0.074 |  | 0.092** |  | 0.085** |  | 0.028 |
|  |  | (0.060) |  | (0.008) |  | (0.006) |  | (0.430) |
| [4] Rural area/village |  | 0.049 |  | 0.11** |  | 0.096** |  | 0.022 |
|  |  | (0.194) |  | (0.001) |  | (0.001) |  | (0.524) |
| Employment, current job |  | 0.29*** |  | 0.21*** |  | 0.027 |  | 0.12*** |
|  |  | (0.000) |  | (0.000) |  | (0.297) |  | (0.000) |
| Self-employment, current job |  | 0.21*** |  | 0.22*** |  | 0.0031 |  | 0.073 |
|  |  | (0.000) |  | (0.000) |  | (0.929) |  | (0.063) |
| [1] Primary school |  | 0.24* |  | 0.41*** |  | -0.025 |  | 0.20* |
|  |  | (0.012) |  | (0.000) |  | (0.721) |  | (0.026) |
| [2] Lower secondary school |  | 0.25** |  | 0.53*** |  | 0.022 |  | 0.29** |
|  |  | (0.008) |  | (0.000) |  | (0.751) |  | (0.001) |
| [3] Upper secondary school |  | 0.26** |  | 0.61*** |  | -0.0095 |  | 0.31*** |
|  |  | (0.006) |  | (0.000) |  | (0.891) |  | (0.001) |
| [4] Post-secondary non-tertiary education |  | 0.37*** |  | 0.65*** |  | 0.057 |  | 0.30** |
|  |  | (0.001) |  | (0.000) |  | (0.472) |  | (0.003) |
| [5] First stage tertiary education |  | 0.30** |  | 0.65*** |  | -0.0024 |  | 0.29** |
|  |  | (0.002) |  | (0.000) |  | (0.973) |  | (0.002) |
| [6] Second stage tertiary education |  | 0.39** |  | 0.72*** |  | -0.089 |  | 0.23 |
|  |  | (0.004) |  | (0.000) |  | (0.393) |  | (0.079) |
| [1] Fair |  | 1.06*** |  | 1.13*** |  | 0.10** |  | 1.22*** |
|  |  | (0.000) |  | (0.000) |  | (0.002) |  | (0.000) |
| [2] Good |  | 1.49*** |  | 1.76*** |  | 0.098** |  | 1.86*** |
|  |  | (0.000) |  | (0.000) |  | (0.004) |  | (0.000) |
| [3] Very good |  | 1.81*** |  | 2.11*** |  | 0.19*** |  | 2.13*** |
|  |  | (0.000) |  | (0.000) |  | (0.000) |  | (0.000) |
| [4] Excellent |  | 2.08*** |  | 2.42*** |  | 0.30*** |  | 2.22*** |
|  |  | (0.000) |  | (0.000) |  | (0.000) |  | (0.000) |
| Drugs for depression |  | -0.43*** |  | -0.58*** |  | -0.081* |  | -1.21*** |
|  |  | (0.000) |  | (0.000) |  | (0.013) |  | (0.000) |
| [1] Middle income |  | 0.23*** |  | 0.25*** |  | 0.026 |  | 0.14*** |
|  |  | (0.000) |  | (0.000) |  | (0.394) |  | (0.000) |
| [2] Upper middle income |  | 0.30*** |  | 0.31*** |  | 0.021 |  | 0.17*** |
|  |  | (0.000) |  | (0.000) |  | (0.494) |  | (0.000) |
| [3] High income |  | 0.26*** |  | 0.29*** |  | 0.038 |  | 0.12*** |
|  |  | (0.000) |  | (0.000) |  | (0.166) |  | (0.001) |
| _cons | 4.79*** | 1.50* | 2.37*** | -1.00 | 6.84*** | 6.61*** | 3.71*** | 1.85** |
|  | (0.000) | (0.014) | (0.000) | (0.062) | (0.000) | (0.000) | (0.000) | (0.001) |
| N | 22018 | 19941 | 21359 | 19390 | 22121 | 20021 | 21882 | 19815 |
| R² | 0.13 | 0.25 | 0.19 | 0.37 | 0.17 | 0.17 | 0.066 | 0.28 |
| adjusted R² | 0.13 | 0.25 | 0.19 | 0.37 | 0.17 | 0.17 | 0.065 | 0.28 |
