# Supplementary material for: Marriage, parenthood and social network: Subjective well-being and mental health in old age
Source: PLoS One. 2019 Jul 24;14(7):e0218704. doi: 10.1371/journal.pone.0218704 (PMC6656342; doi:10.1371/journal.pone.0218704)
Supplement: S14 Table — (DOCX) [file pone.0218704.s019.docx]

**S14 Table. Regressing well-being and mental health on network types controlling for network size, relational dynamics and family status for all countries, female respondents with social support network**

|  | Life satisfaction | | Quality of life (CASP-12) | | Network satisfaction | | Lack of depressive symptoms (EURO-D) | |
| --- | --- | --- | --- | --- | --- | --- | --- | --- |
|  | A | B | A | B | A | B | A | B |
| [2] Children | -0.32*** | -0.19*** | -0.44*** | -0.26*** | 0.21*** | 0.18*** | -0.47*** | -0.20*** |
|  | (0.000) | (0.000) | (0.000) | (0.000) | (0.000) | (0.000) | (0.000) | (0.000) |
| [3] Other Relatives | -0.20*** | -0.11* | -0.17*** | -0.063 | 0.20*** | 0.18*** | -0.43*** | -0.26*** |
|  | (0.000) | (0.019) | (0.000) | (0.138) | (0.000) | (0.000) | (0.000) | (0.000) |
| [4] Family | -0.17*** | -0.082 | -0.24*** | -0.12** | 0.18*** | 0.16*** | -0.32*** | -0.12** |
|  | (0.000) | (0.052) | (0.000) | (0.002) | (0.000) | (0.000) | (0.000) | (0.007) |
| [5] Friends | -0.16*** | -0.11* | -0.069 | 0.028 | 0.25*** | 0.23*** | -0.41*** | -0.24*** |
|  | (0.001) | (0.020) | (0.111) | (0.502) | (0.000) | (0.000) | (0.000) | (0.000) |
| [6] Diverse | -0.26*** | -0.11* | -0.28*** | -0.093* | 0.16*** | 0.14*** | -0.57*** | -0.31*** |
|  | (0.000) | (0.031) | (0.000) | (0.038) | (0.000) | (0.000) | (0.000) | (0.000) |
| Size of social network | 0.11*** | 0.085*** | 0.12*** | 0.084*** | 0.075*** | 0.075*** | 0.060*** | 0.024** |
|  | (0.000) | (0.000) | (0.000) | (0.000) | (0.000) | (0.000) | (0.000) | (0.003) |
| Average contact 0-6 | 0.069*** | 0.075*** | 0.082*** | 0.078*** | 0.18*** | 0.18*** | 0.036* | 0.039* |
|  | (0.000) | (0.000) | (0.000) | (0.000) | (0.000) | (0.000) | (0.033) | (0.012) |
| Average closeness 0-3 | 0.34*** | 0.29*** | 0.31*** | 0.26*** | 0.69*** | 0.67*** | 0.17*** | 0.12*** |
|  | (0.000) | (0.000) | (0.000) | (0.000) | (0.000) | (0.000) | (0.000) | (0.000) |
| Average proximity 0-5 | -0.036** | -0.0026 | -0.074*** | -0.031** | -0.054*** | -0.058*** | -0.038** | 0.017 |
|  | (0.007) | (0.847) | (0.000) | (0.009) | (0.000) | (0.000) | (0.006) | (0.204) |
| Married/registered partnership | 0.43*** | 0.30*** | 0.19*** | 0.13** | -0.045** | 0.016 | 0.15*** | -0.057 |
|  | (0.000) | (0.000) | (0.000) | (0.008) | (0.008) | (0.704) | (0.000) | (0.314) |
| [1] Having 1 child | -0.091* | -0.088 | 0.024 | 0.020 | 0.041 | 0.022 | -0.12* | -0.070 |
|  | (0.045) | (0.061) | (0.582) | (0.630) | (0.197) | (0.528) | (0.016) | (0.145) |
| [2] Having 2 children | 0.051 | 0.045 | 0.094* | 0.067 | 0.065* | 0.042 | 0.023 | 0.056 |
|  | (0.239) | (0.322) | (0.025) | (0.110) | (0.034) | (0.217) | (0.629) | (0.235) |
| [3] Having 3 or more children | -0.022 | -0.0067 | 0.019 | 0.018 | 0.063 | 0.040 | -0.064 | 0.00077 |
|  | (0.659) | (0.897) | (0.694) | (0.699) | (0.064) | (0.291) | (0.232) | (0.988) |
| Number of resident children | -0.050* | -0.083*** | -0.089*** | -0.13*** | -0.027* | -0.027* | -0.0047 | -0.029 |
|  | (0.011) | (0.000) | (0.000) | (0.000) | (0.027) | (0.039) | (0.824) | (0.148) |
| Number of grandchildren | -0.0071 | 0.0032 | -0.017*** | -0.00072 | 0.0096** | 0.0096** | -0.024*** | -0.0072 |
|  | (0.157) | (0.513) | (0.000) | (0.863) | (0.002) | (0.003) | (0.000) | (0.138) |
| **Controls** |  |  |  |  |  |  |  |  |
| Age at interview | 0.0066 | 0.022 | 0.11*** | 0.12*** | 0.015 | 0.011 | 0.11*** | 0.11*** |
|  | (0.643) | (0.142) | (0.000) | (0.000) | (0.109) | (0.268) | (0.000) | (0.000) |
| Age at interview, squared | 0.0000036 | 0.0000092 | -0.00093*** | -0.00090*** | -0.000095 | -0.000070 | -0.00092*** | -0.00071*** |
|  | (0.972) | (0.933) | (0.000) | (0.000) | (0.157) | (0.345) | (0.000) | (0.000) |
| sh_country==[2]BEL | -0.43*** | -0.29*** | -0.66*** | -0.48*** | -0.30*** | -0.30*** | -0.52*** | -0.24*** |
|  | (0.000) | (0.000) | (0.000) | (0.000) | (0.000) | (0.000) | (0.000) | (0.000) |
| sh_country==[3]CHE | 0.33*** | 0.14** | 0.56*** | 0.33*** | 0.17*** | 0.19*** | 0.066 | -0.12* |
|  | (0.000) | (0.006) | (0.000) | (0.000) | (0.000) | (0.000) | (0.222) | (0.027) |
| sh_country==[4]CZE | -0.83*** | -0.52*** | -1.24*** | -0.87*** | -0.062 | -0.074 | -0.25*** | 0.093 |
|  | (0.000) | (0.000) | (0.000) | (0.000) | (0.066) | (0.053) | (0.000) | (0.065) |
| sh_country==[5]DEU | -0.31*** | -0.25*** | 0.0034 | 0.099 | 0.079 | 0.086 | -0.26*** | -0.16* |
|  | (0.000) | (0.000) | (0.958) | (0.107) | (0.119) | (0.109) | (0.000) | (0.017) |
| sh_country==[6]DNK | 0.49*** | 0.27*** | 0.46*** | 0.21*** | 0.35*** | 0.34*** | 0.20** | -0.0061 |
|  | (0.000) | (0.000) | (0.000) | (0.000) | (0.000) | (0.000) | (0.001) | (0.919) |
| sh_country==[7]ESP | -0.78*** | -0.38*** | -1.16*** | -0.60*** | -0.19*** | -0.19*** | -1.03*** | -0.44*** |
|  | (0.000) | (0.000) | (0.000) | (0.000) | (0.000) | (0.000) | (0.000) | (0.000) |
| sh_country==[8]EST | -1.22*** | -0.90*** | -0.87*** | -0.49*** | 0.16*** | 0.24*** | -0.85*** | -0.35*** |
|  | (0.000) | (0.000) | (0.000) | (0.000) | (0.000) | (0.000) | (0.000) | (0.000) |
| sh_country==[9]FRA | -0.84*** | -0.67*** | -0.43*** | -0.15*** | -0.13*** | -0.14*** | -0.70*** | -0.37*** |
|  | (0.000) | (0.000) | (0.000) | (0.001) | (0.000) | (0.000) | (0.000) | (0.000) |
| sh_country==[10]HUN | -1.52*** | -0.96*** | -1.27*** | -0.61*** | 0.024 | 0.061 | -1.05*** | -0.41*** |
|  | (0.000) | (0.000) | (0.000) | (0.000) | (0.525) | (0.148) | (0.000) | (0.000) |
| sh_country==[11]ITA | -0.60*** | -0.39*** | -1.57*** | -1.30*** | -0.013 | -0.021 | -0.66*** | -0.37*** |
|  | (0.000) | (0.000) | (0.000) | (0.000) | (0.711) | (0.570) | (0.000) | (0.000) |
| sh_country==[12]NLD | -0.25*** | -0.26*** | 0.32*** | 0.32*** | -0.49*** | -0.51*** | -0.0047 | 0.064 |
|  | (0.000) | (0.000) | (0.000) | (0.000) | (0.000) | (0.000) | (0.936) | (0.244) |
| sh_country==[13]POL | -0.79*** | -0.26*** | -1.00*** | -0.40*** | 0.15** | 0.19*** | -1.12*** | -0.54*** |
|  | (0.000) | (0.001) | (0.000) | (0.000) | (0.002) | (0.000) | (0.000) | (0.000) |
| sh_country==[14]PRT | -1.39*** | -0.64*** | -2.14*** | -1.27*** | 0.075 | 0.19*** | -1.47*** | -0.47*** |
|  | (0.000) | (0.000) | (0.000) | (0.000) | (0.089) | (0.000) | (0.000) | (0.000) |
| sh_country==[15]SVN | -0.61*** | -0.37*** | 0.050 | 0.38*** | 0.061 | 0.092* | -0.36*** | -0.11 |
|  | (0.000) | (0.000) | (0.369) | (0.000) | (0.147) | (0.038) | (0.000) | (0.081) |
| sh_country==[16]SWE | 0.24*** | 0.15** | 0.084 | -0.0020 | 0.28*** | 0.25*** | 0.11 | 0.086 |
|  | (0.000) | (0.009) | (0.134) | (0.970) | (0.000) | (0.000) | (0.089) | (0.159) |
| Divorced/living separated |  | -0.17** |  | -0.10 |  | -0.00083 |  | -0.13* |
|  |  | (0.005) |  | (0.061) |  | (0.986) |  | (0.043) |
| Widowed |  | 0.035 |  | 0.12* |  | 0.11* |  | -0.12* |
|  |  | (0.555) |  | (0.026) |  | (0.014) |  | (0.040) |
| [1] Suburbs of big city |  | -0.0068 |  | 0.026 |  | 0.014 |  | -0.11* |
|  |  | (0.870) |  | (0.478) |  | (0.629) |  | (0.010) |
| [2] Large town |  | 0.020 |  | 0.0073 |  | 0.062* |  | -0.092* |
|  |  | (0.592) |  | (0.829) |  | (0.022) |  | (0.018) |
| [3] Small town |  | 0.098** |  | 0.044 |  | 0.047 |  | -0.016 |
|  |  | (0.006) |  | (0.159) |  | (0.065) |  | (0.654) |
| [4] Rural area/village |  | 0.065 |  | 0.039 |  | 0.0066 |  | -0.033 |
|  |  | (0.065) |  | (0.199) |  | (0.784) |  | (0.344) |
| Employment, current job |  | 0.085** |  | 0.13*** |  | -0.0057 |  | 0.071* |
|  |  | (0.006) |  | (0.000) |  | (0.789) |  | (0.026) |
| Self-employment, current job |  | 0.10* |  | 0.088 |  | -0.091* |  | 0.093 |
|  |  | (0.044) |  | (0.072) |  | (0.034) |  | (0.102) |
| [1] Primary school |  | 0.013 |  | 0.34*** |  | -0.068 |  | 0.25** |
|  |  | (0.869) |  | (0.000) |  | (0.155) |  | (0.001) |
| [2] Lower secondary school |  | 0.053 |  | 0.41*** |  | -0.10* |  | 0.33*** |
|  |  | (0.493) |  | (0.000) |  | (0.042) |  | (0.000) |
| [3] Upper secondary school |  | 0.084 |  | 0.50*** |  | -0.11* |  | 0.48*** |
|  |  | (0.281) |  | (0.000) |  | (0.030) |  | (0.000) |
| [4] Post-secondary non-tertiary education |  | 0.12 |  | 0.65*** |  | -0.11 |  | 0.62*** |
|  |  | (0.176) |  | (0.000) |  | (0.059) |  | (0.000) |
| [5] First stage tertiary education |  | 0.19* |  | 0.55*** |  | -0.15** |  | 0.53*** |
|  |  | (0.016) |  | (0.000) |  | (0.003) |  | (0.000) |
| [6] Second stage tertiary education |  | 0.41** |  | 0.68*** |  | -0.039 |  | 0.58*** |
|  |  | (0.005) |  | (0.000) |  | (0.696) |  | (0.000) |
| [1] Fair |  | 0.98*** |  | 1.09*** |  | 0.11*** |  | 1.25*** |
|  |  | (0.000) |  | (0.000) |  | (0.000) |  | (0.000) |
| [2] Good |  | 1.50*** |  | 1.78*** |  | 0.13*** |  | 2.01*** |
|  |  | (0.000) |  | (0.000) |  | (0.000) |  | (0.000) |
| [3] Very good |  | 1.81*** |  | 2.15*** |  | 0.21*** |  | 2.37*** |
|  |  | (0.000) |  | (0.000) |  | (0.000) |  | (0.000) |
| [4] Excellent |  | 2.14*** |  | 2.45*** |  | 0.34*** |  | 2.50*** |
|  |  | (0.000) |  | (0.000) |  | (0.000) |  | (0.000) |
| Drugs for depression |  | -0.51*** |  | -0.61*** |  | -0.099*** |  | -1.15*** |
|  |  | (0.000) |  | (0.000) |  | (0.000) |  | (0.000) |
| [1] Middle income |  | 0.099** |  | 0.14*** |  | -0.011 |  | 0.051 |
|  |  | (0.005) |  | (0.000) |  | (0.656) |  | (0.172) |
| [2] Upper middle income |  | 0.17*** |  | 0.13*** |  | 0.0029 |  | 0.0057 |
|  |  | (0.000) |  | (0.000) |  | (0.905) |  | (0.874) |
| [3] High income |  | 0.21*** |  | 0.20*** |  | -0.064** |  | 0.035 |
|  |  | (0.000) |  | (0.000) |  | (0.007) |  | (0.302) |
| _cons | 6.32*** | 3.68*** | 3.57*** | 0.12 | 5.75*** | 5.85*** | 4.65*** | 1.81*** |
|  | (0.000) | (0.000) | (0.000) | (0.795) | (0.000) | (0.000) | (0.000) | (0.001) |
| N | 28606 | 25650 | 27598 | 24824 | 28748 | 25749 | 28444 | 25501 |
| R² | 0.14 | 0.25 | 0.22 | 0.39 | 0.16 | 0.17 | 0.083 | 0.29 |
| adjusted R² | 0.14 | 0.25 | 0.22 | 0.39 | 0.16 | 0.16 | 0.082 | 0.29 |
